# p63 affects distinct metabolic pathways during keratinocyte senescence, evaluated by metabolomic profile and gene expression analysis

**DOI:** 10.1038/s41419-024-07159-7

**Published:** 2024-11-14

**Authors:** Maria Cristina Piro, Rosalba Pecorari, Artem Smirnov, Angela Cappello, Erica Foffi, Anna Maria Lena, Yufang Shi, Gerry Melino, Eleonora Candi

**Affiliations:** 1 Department of Experimental Medicine, TOR, University of Rome “Tor Vergata”, Rome, Italy; 2 IDI-IRCCS, Rome, Italy; 3 Interdisciplinary Department of Medicine, University of Bari “Aldo Moro”, Bari, Italy; 4 The Fourth Affiliated Hospital of Soochow University, Institutes for Translational Medicine, State Key Laboratory of Radiation Medicine and Protection, Key Laboratory of Stem Cells and Medical Biomaterials of Jiangsu Province, Medical College of Soochow University, Soochow University, Suzhou, China

**Keywords:** Metabolomics, Transcription

## Abstract

Unraveling the molecular nature of skin aging and keratinocyte senescence represents a challenging research project in epithelial biology. In this regard, depletion of p63, a p53 family transcription factor prominently expressed in human and mouse epidermis, accelerates both aging and the onset of senescence markers in vivo animal models as well as in ex vivo keratinocytes. Nonetheless, the biochemical link between p63 action and senescence phenotype remains largely unexplored. In the present study, through ultrahigh performance liquid chromatography–tandem mass spectroscopy (UPLC–MS/MS) and gas chromatography/mass spectrometry (GC/MS) metabolomic analysis, we uncover interesting pathways linking replicative senescence to metabolic alterations during p63 silencing in human keratinocytes. Integration of our metabolomic profiling data with targeted transcriptomic investigation empowered us to demonstrate that absence of p63 and senescence share similar modulation profiles of oxidative stress markers, pentose phosphate pathway metabolites and lyso-glycerophospholipids, the latter due to enhanced phospholipases gene expression profile often under p63 direct/indirect gene control. Additional biochemical features identified in deranged keratinocytes include a relevant increase in lipids production, glucose and pyruvate levels as confirmed by upregulation of gene expression of key lipid synthesis and glycolytic enzymes, which, together with improved vitamins uptake, characterize senescence phenotype. Silencing of p63 in keratinocytes instead, translates into a blunted flux of metabolites through both glycolysis and the Krebs cycle, likely due to a p63-dependent reduction of hexokinase 2 and citrate synthase gene expression. Our findings highlight the potential role of p63 in counteracting keratinocyte senescence also through fine regulation of metabolite levels and relevant biochemical pathways. We believe that our research might contribute significantly to the discovery of new implications of p63 in keratinocyte senescence and related diseases.

## Introduction

Originally demonstrated in fibroblasts by Hayflick and Moohead in 1961, senescence is defined as a form of permanent and irreversible cell cycle arrest [1]. This finite cell duplication capacity is a peculiar cell response to a variety of stress-inducing stimuli such as telomere dysfunction, persistent DNA damage, mitochondrial stress and oncogene activation. The senescent-associated secretory phenotype (SASP) allows senescent cells to affect the surrounding microenvironment. The dynamic composition of SASP includes pro-inflammatory cytokines, bioactive lipids, chemokines, damage-associated molecular pattern (DAMPS), grow factors and extracellular matrix proteases [2, 3]. Depending on the cellular and physiological context, senescence and its secretory activity can have favorable outcomes such as protecting young organisms from cancer development, enhancing embryogenesis or favoring tissue repair and regeneration. Alternatively, harmful effects of senescent cells are due to their increase and persistence in tissues of aged mammals where SASP sustains its implication in age-related pathologies such as cancer, chronic inflammation or neurodegeneration [4–7]. TP63, together with TP53 and TP73, belongs to the p53 family of transcription factors [8–13]. The TP63 gene expands its coding potential by differential use of two alternative promoters/start sites, which generates two isoforms, named TAp63 and $\Delta$Np63, depending on whether they include or lack the N-terminal transactivation domain [14]. Furthermore, alternative splicing at exons in the 3′ portion of the p63 gene provides three (α, β, and γ) different TA and $\Delta$Np63 proteins [15].

While the $\Delta$Np63α isoform is specifically considered to be the key regulator of proliferation and stemness in stratified epithelia such as epidermis [16], several lines of evidence have shown that depletion of either the $\Delta$Np63 or TAp63 isoform induces the onset of senescence markers in mouse skin [17–19], although the role of TAp63 is highly debated [20]. As a master of epithelial cell proliferation, differentiation and survival, p63 is also involved in lifespan and longevity, and it is not surprising that p63 heterozygous mice or conditional knockout p63 display, in addition to the senescence phenotype, also characteristics of accelerated aging and shortened lifespan [17, 18]. In this last regard, $\Delta$Np63 represses senescence in primary human keratinocytes due to the direct downregulation of microRNAs involved in the negative control of the pro-survival sirtuin SIRT1 [21]. Because the proliferative capacity and metabolism of cells are closely interconnected, p63 also exerts its action in the control of some important biochemical pathways, including glucose metabolism [22], glutamine [23], antioxidant defense [24, 25] lipid metabolism [26], nucleotide metabolism [27], serine and one-carbon metabolism [28].

Although in the absence of cell division, the senescent state is characterized by extensive metabolic reprogramming necessary to define and maintain the senescent phenotype which is still metabolically active [29, 30]. The attractive position of p63, at the crossroad between control of cell proliferation, aging, and involvement in important metabolic pathways, inspired us to investigate the biochemical metabolic alterations that occur when senescent keratinocytes undergo senescence or p63 silencing. Indeed, like most somatic cells in culture, normal human epidermal keratinocytes (NHEK) also exhibit a limited number of doublings that eventually ends in senescence [31, 32].

Here, we investigate the targeted biochemical landscape of human replicative senescent keratinocytes compared to p63-depleted keratinocytes, with the aim of describing the metabolic similarities and differences between these two sets of intrinsically deranged cells and thus their biochemical contribution to skin aging. By using global metabolomic analyses, integrated with gene expression analyses on high-throughput ChIP-seq data, we show how p63 inhibits senescence by regulating levels of reactive oxygen species (ROS), lipid metabolism and pentose phosphate pathway (PPP).

## Results

### Senescence or p63 loss in keratinocytes leads to oxidative stress

**Fig. 1:**
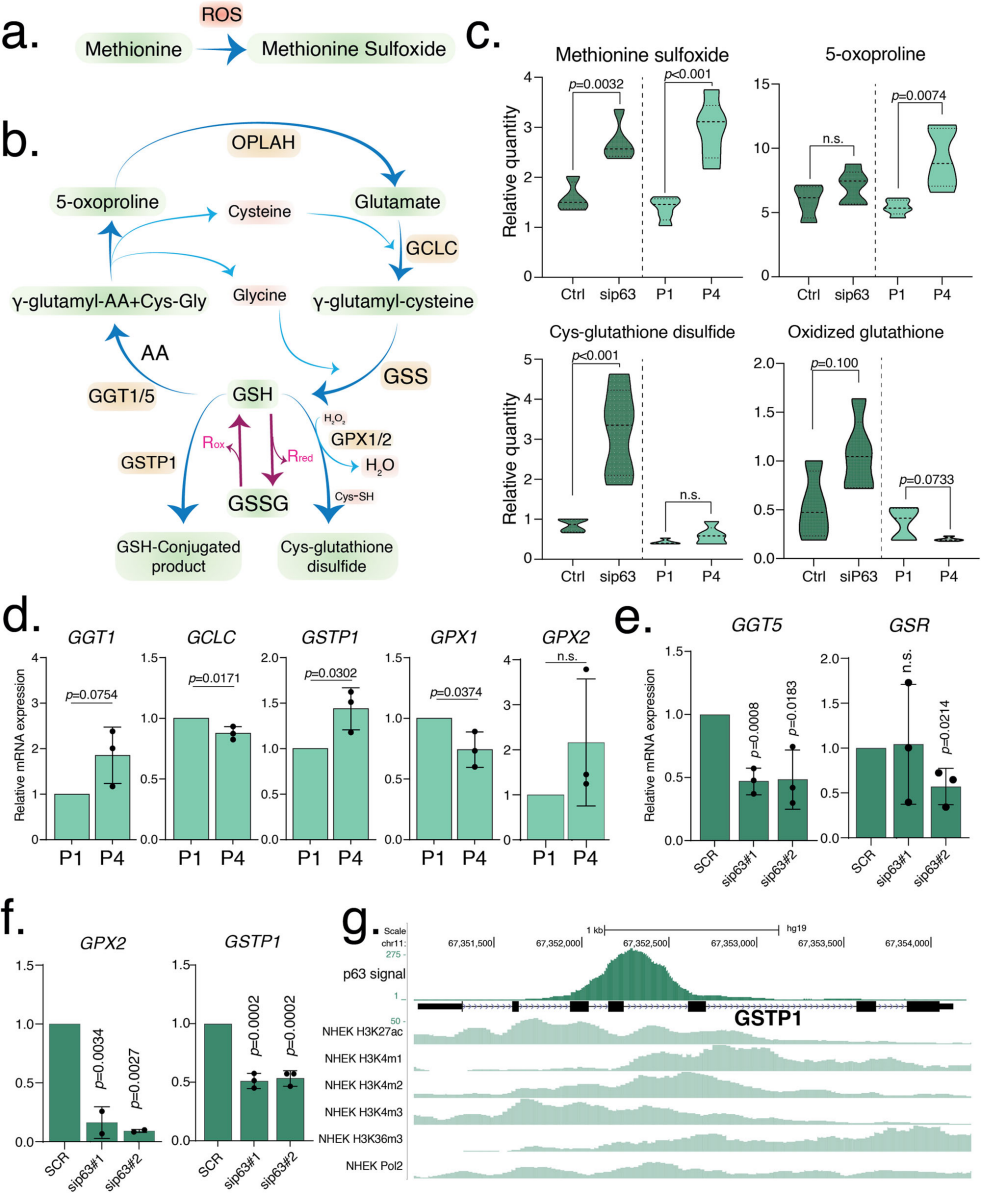
Increased oxidative stress. **a** Methionine oxidation involving reactive oxygen species (ROS). **b** Graphical representation of the glutathione pathway. **c** Relative quantity of methionine sulfoxide, 5-oxoproline, cys-glutathione disulfide and GSSG in senescent (P1, P4) and transfected (Ctrl, sip63 siRNAs) keratinocytes. Data are shown as mean ± SD of *N* = 5 biological replicates. The adjusted *p* values were calculated using Student’s *t*-test. **d** The mRNA expression level of *GGT1, GCLC, GSTP1, GPX1* and *GPX2* were evaluated using qRT-PCR in senescent (P1, P4) keratinocytes. Data are shown as mean ± SD of *N* = 3 biological replicates. The adjusted *p* values were calculated using Student’s *t*-test. n.s. = non-significant. **e** The mRNA expression level of *GGT5*, and *GSR* were evaluated using qRT-PCR in transfected (Ctrl, sip63#1 and sip63#2 siRNAs) keratinocytes. Data are shown as mean ± SD of *N* = 3 biological replicates. The adjusted *p* values were calculated using Student’s *t*-test. n.s. = non-significant. **f** The mRNA expression level of *GPX2* and *GSTP1* were evaluated using qRT-PCR in transfected (Ctrl, sip63#1 and sip63#2 siRNAs) keratinocytes. Data are shown as mean ± SD of *N* = 3 biological replicates. The adjusted *p* values were calculated using Student’s *t*-test. **g** ChIP-seq signals profiles of p63, H3K27Ac, H3K4me1, H3K4me2, H3K4me3, H3K36me3 and Polymerase II (range 1–50) in NHEK at *GSTP1* gene region.

The protective role of p63 against oxidative stress has been already preliminarily investigated [24, 25, 28], showing that senescent cells produce excessive ROS, leading to oxidative stress [33]. In this study, we investigated the metabolic changes related to oxidative stress in both p63-depleted (“Ctrl” vs “sip63”) or senescent (passage 1 to 4, “P4” vs “P1”) keratinocytes. Characterization of our cultured human keratinocytes cellular model confirmed the presence of ΔNp63 isoform, which decreased during senescence, while TAp63 isoform was undetected at protein level. In p63-silenced keratinocytes, both TAp63 and ΔNp63 protein isoforms were undetected (Fig. S1a–d). Our data indicate an increase in metabolites related to oxidative stress in both p63-KD and P4 senescent keratinocytes compared to controls (Figs. 1a–c and 5). Accordingly, methionine and cysteine are particularly prone to reversible oxidation by excess ROS to produce methionine sulfoxide and cysteine glutathione disulfide products, respectively [34]. Indeed, we detected increasing methionine sulfoxide levels that increased significantly both in sip63 and in senescent P4 keratinocytes (1.69-fold, *P* = 0.0032 and 2.14-fold, *P* < 0.001, respectively), compared to controls (Figs. 1a–c and 5). Moreover, cysteine glutathione disulfide is overproduced in the absence of p63 (3.75-fold; *P* < 0.001), possibly due to the need to protect protein thiols in oxidative cell context by forming a disulfide bond with glutathione [35]. This agrees with the increased levels of oxidized glutathione (GSSG) in sip63 keratinocytes, although with a lesser significance (1.98-fold, 0.1 > *P* > 0.05) (Figs. 1c and 5). However, the higher levels of GSSG detected in sip63 cells could also be due, in addition to a general oxidative environment, to a significant downregulation of the glutathione reductase (*GSR*) expression (0.57-fold, *P* = 0.0214), as evidenced by its mRNA levels in sip63 (Fig. 1b, e and Table 1).

**Table 1:**
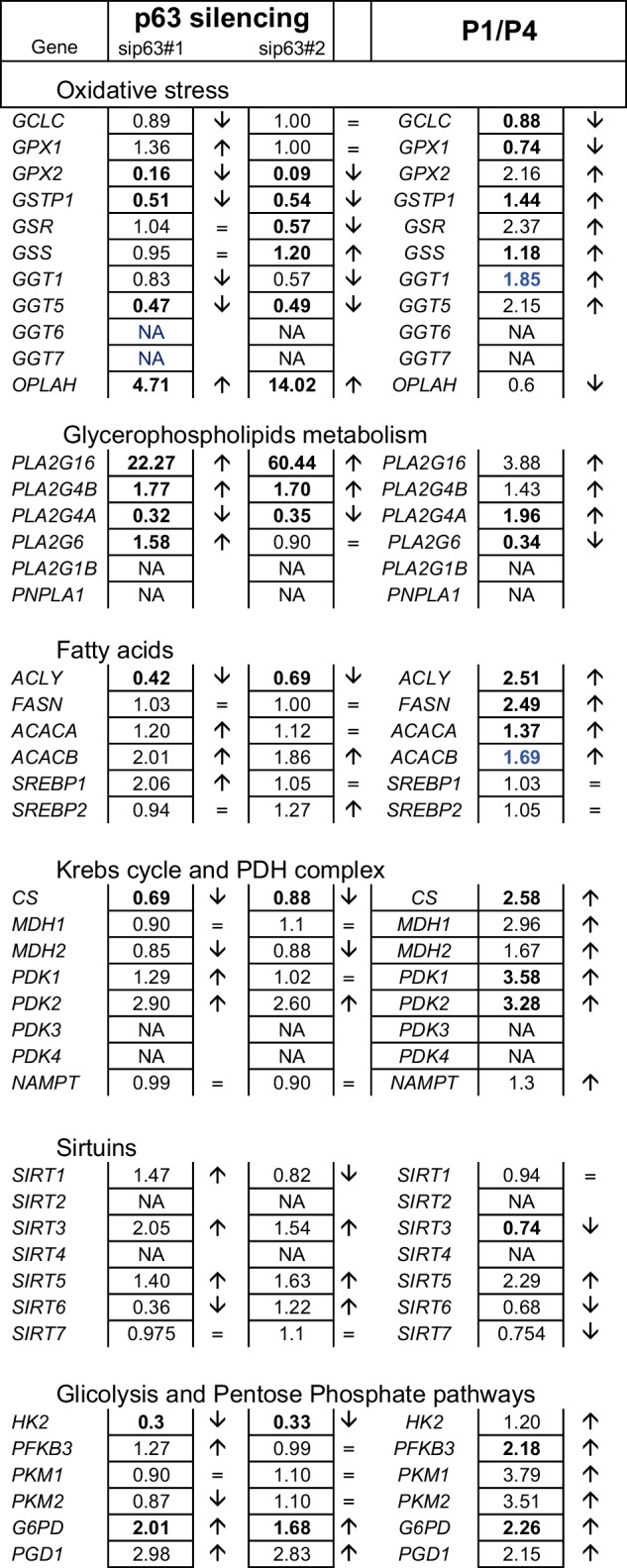
Relative mRNA expression.

Relative mRNA expression of genes implicated into the indicated metabolic pathways, assessed in sip63 keratinocytes vs control with two siRNA, and in P4 senescent vs P1 control keratinocytes.

Values in black/bold indicate statistically significant (*P* ≤ 0.05) decreases or increases in fold of change. Values indicated in blue/bold indicate a trend (0.1 ≥ *P* ≥ 0.05).

In senescent keratinocytes, oxidative stress appears to be less pronounced as no differences in cysteine glutathione disulfide levels were detected in P4 vs P1 while GSSG was lower in P4 compared with P1 (Figs. 1c and 5). Indeed, the expression of *GSR* in P4 senescent cells was consistent with controls P1 (Fig. S1f). Of note, the levels of reduced glutathione (GSH) in both sets of cells (sip63 or senescent), were below the limit of detection of the metabolomics analysis technique used here (see “Materials and Methods” and “Discussion” sections). Low levels of GSH are unlikely due to transcriptional downregulation of the enzymes necessary to its de novo synthesis, i.e., *GCLC* (glutamate cysteine ligase) and *GSS* (glutathione synthetase), whose mRNA levels were not significantly altered in sip63 or senescent keratinocytes compared to controls, (Fig. 1b, d and Fig. S1e, f). Possibly, keratinocytes per se have a high GSH turnover, as referred to in the “Discussion” section [36]. 5-Oxoprolin, one of the main products of the “glutathione cycle” which breaks down glutathione into its constituent amino acids [37], can lead to generation of reactive species and cellular oxidative stress when present at high levels [38, 39]. In our analysis, 5-oxoproline is, indeed, significantly increased in P4 senescent keratinocytes (1.70-fold, *P* = 0.0074) and tends to increase in sip63 keratinocytes although below threshold of the statistical significance (Figs. 1c and 5). The production of 5-oxoproline is derived from the activity of the ecto-enzyme γ-glutamyl peptidase (GGT) that breaks down GSH to form γ-glutamyl amino acid and the dipeptide cysteinylglycine. The γ-glutamyl amino acid is then converted intracellularly into 5-oxoproline and free aa (Fig. 1b). We analyzed the expression levels of four GGT isoforms, namely, *GGT1*, *GGT5*, *GGT6* and *GGT7*. The mRNA of the last two isoenzymes (*GGT6* or *GGT7*) were not detectable in human keratinocytes. However, we observed a notable modulation of both *GGT1* and *GGT5* mRNA whose proteins are also reported to be the only catalytically active ones [40]. In senescence, *GGT1* mRNA was found to be upregulated in P4 keratinocytes (1.85-fold, *P* = 0.0754) (Fig. 1D and Table 1) while *GGT5* mRNA levels are unchanged in the same cells compared to controls (Fig. S1f and Table 1). The less pronounced increase in 5-oxoproline in sip63 could be due to a marked and significant 50% downregulation of *GGT5* mRNA in sip63 keratinocytes (0.47-fold, *P* = 0.0008) (Fig. 1e and Table 1) while *GGT1* levels are consistent in the same cells, respect to controls (Fig. S1 and Table 1). However, steady-state levels of 5-oxoproline could also depend on its rate of degradation by 5-oxoprolinase (OPLAH) (Fig. 1a) [38]. Indeed, in agreement with our metabolomic results, *OPLAH* mRNA level was found upregulated in p63-silenced cells (14.04-fold, *P* = 0.0002) while no changes in senescence were identified (Fig. S1b and Table 1).

We observed modulation of mRNA expression of other enzymes that utilize reduced glutathione as substrate, such as glutathione peroxidases (*GPX1*, *GPX2*), and glutathione *S*-transferase (*GSTP1*). GPX1 and GPX2 belong to the glutathione peroxidase family of enzymes that use the reducing power of GSH to detoxify cells from dangerous pro-oxidant molecules such as organic hydroperoxides and hydrogen peroxide [41]. Both p63 silencing and senescence affect the mRNA levels of these two enzymes, indicating a re-adjustment of antioxidant defense in both metabolically altered cell groups. As a consequence of the increasing oxidative stress and probably of the low level of the GSH as substrate, *GPX1* mRNA levels are significantly negatively regulated in senescence (0.74-fold, *P* = 0.0374) while *GPX2* mRNA is doubled, although not statistically significant (Fig. 1d and Table 1). It is worth mentioning that GPX1 protein can be regulated also by phosphorylation by c-Abl and Arg tyrosine kinases [42]. No variations were found in sip63 vs controls for *GPX1* (Fig. S1e and Table 1), while *GPX2* is a well-known antioxidant gene regulated by p63 [43], and was found downregulated upon p63 depletion also in our system (0.09-fold, *P* = 0.0027) (Fig. 1f and Table 1).

The third modulated enzyme is GSTP1, a member of the glutathione *S*-transferases enzyme family. These set of enzymes conjugate reduced glutathione to many hydrophobic and electrophilic compounds for detoxification purposes [44]. *GSTP1* expression is markedly and significantly downregulated by 50% in sip63 keratinocytes (0.51-fold, *P* = 0.0002; Fig. 1f and Table 1). We hypothesize that this is as a direct effect of p63 silencing, as a very pronounced signal for p63 binding across intron 4 on the *GSTP1* gene is evident from p63 ChIP-seq analysis in proliferating keratinocytes (GSE59827), in a genomic region of open chromatin state and pol II binding (Fig. 1g) [45]. *GSTP1* mRNA level in senescence P4 keratinocytes is instead upregulated (1.44-fold, *P* = 0.0302; Fig. 1d and Table 1).

Altogether, both senescent and sip63 keratinocytes exhibit metabolomic signatures of elevated oxidative stress. While increased level of methionine sulfoxide was found in both senescent and sip63 cells, 5-oxoproline and cysteine glutathione disulfide were elevated in only senescent or sip63 keratinocytes, respectively. At the transcript levels, the modulations of the genes involved in oxidative stress show a significative downregulation of the glutathione circuit of antioxidant defense in sip63 cells compared to senescent cells. In fact, *GGT5*, *GSTP1*, *GSR*, and *GPX2* are drastically downregulated in p63 silencing, while *GGT1*, *GSTP1*, and *GPX2* are upregulated in senescence.

### Glycerophospholipid metabolism is deregulated in senescent cells or after p63 depletion

**Fig. 2:**
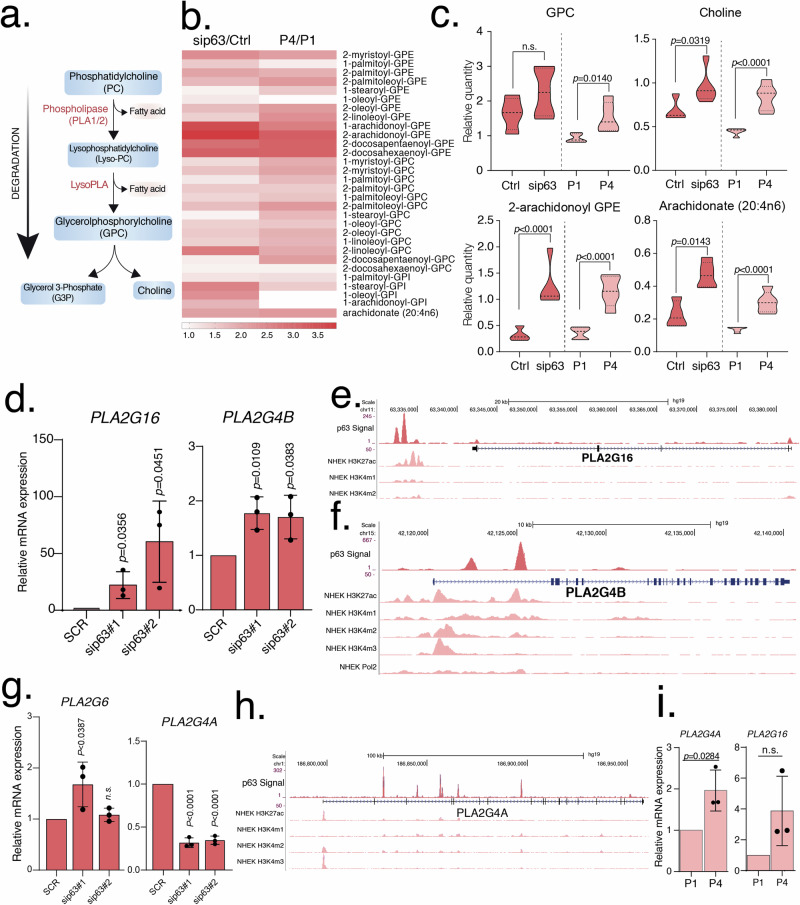
Altered glycerophospholipid metabolism. **a** Graphical representation of the glycerophospholipids degradation. **b** Heatmap shows the relative quantity of lysophospholipids in senescent (P1, P4) and transfected (Ctrl, sip63 siRNAs) keratinocytes. Data are shown as mean of *N* = 5 biological replicates. **c** Relative quantity of GPC, choline, 2-arachidonoyl GPE and arachidonate (20:4n6) in senescent (P1, P4) and transfected (Ctrl, sip63 siRNAs) keratinocytes. Data are shown as mean ± SD of *N* = 5 biological replicates. The adjusted *p* values were calculated using Student’s *t*-test. **d** The mRNA expression levels of *PLA2G16* and *PLA2G4B* were evaluated using qRT-PCR in transfected (Ctrl, sip63#1 and sip63#2 siRNAs) keratinocytes. Data are shown as mean ± SD of *N* = 3 biological replicates. The adjusted *p* values were calculated using Student’s *t*-test. **e** ChIP-seq signals profiles of p63, H3K27Ac, H3K4me1 and H3K4me2 (range 1–50) in NHEK at *PLA2G16* gene region. **f** ChIP-seq signals profiles of p63, H3K27Ac, H3K4me1, H3K4me2, H3K4me3 and Polymerase II (range 1–50) in NHEK at *PLA2G4B* gene region. **g** The mRNA expression levels of *PLA2G6* and *PLA2G4A* were evaluated using qRT-PCR in transfected (Ctrl, sip63#1 and sip63#2 siRNAs) keratinocytes. Data are shown as mean ± SD of *N* = 3 biological replicates. The adjusted *p* values were calculated using Student’s *t*-test. **h** ChIP-seq signals profiles of p63, H3K27Ac, H3K4me1, H3K4me2 and H3K4me3 (range 1–50) in NHEK at *PLA2G4A* gene region. **i** The mRNA expression levels of *PLA2G4A* and *PLA2G16* were evaluated using qRT-PCR in senescent (P1, P4) keratinocytes. Data are shown as mean ± SD of *N* = 3 biological replicates. The adjusted *p* values were calculated using Student’s *t*-test. n.s. non-significant.

Phospholipases A1 (PLA1) and A2 (PLA2) are acyl hydrolases that catalyze the removal of fatty acids from the sn1 or sn2 position of membrane glycerophospholipids producing 2-acyl or 1-acyl-lysophospholipids and free fatty acids such as polyunsaturated arachidonic acid [46]. Acyl-lysophospholipids are precursors of powerful lipid mediator and signaling molecules with important roles in cell metabolism [47]. In our experiments, we found a significant increase of 1- and 2-acyl-lysophospholipids in both p63-silenced and P4 senescent keratinocytes, compared to controls (Figs. 2a, b and 5). Glycerophosphocholine (GPC) and glycero-phosphoethanolamine (GPE) are equally present in both 1- and 2-acyl-lysophospholipid while the glycero-phosphoinositol (GPI) anchor is represented only in 1-acyl-lysophospholipids. We also saw a strong enrichment in 1-arachidonoyl-GPE (3.56-fold, *P* = 0.0157 in sip63, and 2.65-fold, *P* = 0.0099 in P4) and 2-arachidonoyl-GPE (3.86-fold, *P* < 0.001 in sip63 and 3.24-fold, *P* < 0.001 in P4) evident in both senescent and sip63 cells. To assess whether elevation in 2-acyl or 1-acyl-lysophospholipids was due to enhanced PLA genes expression, we interrogated mRNA levels of four cytosolic A2 phospholipases: *PLA2G4A*, *PLA2G4B*, *PLA2G6* and *PLAG16A*. The latter has also PLA1 activity. We observed that in either senescence or sip63 conditions, three PLA mRNAs were upregulated and only one was downregulated. Interestingly, we found that keratinocytes express the phospholipase *PLAG16A* (also named *PLAAT3*) (Fig. 2d and Table 1) [48], with a double A1/A2 activity whose mRNA level was strikingly increased from 22 to 60 times in the absence of p63 (22.27–60.44-fold; *P* = 0.0356). Moreover, p63 ChIP-seq analysis (GSE59827) in proliferating human keratinocytes revealed a strong enrichment downstream of the *PLA2G16A* gene (Fig. 2e) in regions of open chromatin state and suggested a possible direct regulation of this gene by p63. A strong upregulating trend of the *PLA2G16A* gene expression was evident also in senescence (3.88-fold), although the strong variability of the senescence experiments did not allow reaching full statistical significance (Fig. 2i and Table 1). *PLA2G4B* (Fig. 2d, f; Fig. S2 and Table 1) was significantly upregulated in p63 silencing (1.70-fold; *P* = 0.0109) and also in senescence, although not statistically significant. *PLA2G6* (Fig. 2g; Fig. S2 and Table 1) was upregulated in sip63 (1.58-fold; *P* = 0.0387) but downregulated in senescent cells (0.34-fold; *P* = 0.0014) while *PLA2G4A* (Fig. 2g–i and Table 1) was significantly increased in senescent cells (1.96-fold, *P* = 0.0284) but downregulated upon p63 silencing (0.33-fold, *P* < 0.0001). In addition, ChIP-seq analysis of *PLA2G4A* and *PLA2G4B* loci revealed several p63 binding sites in regions of open chromatin state and implies a direct regulation p63-mediated (Fig. 2f, h). The mRNAs for the phospholipases *PLA2G1B* and *PNPLA1* were not detected in keratinocytes (Table 1).

Previous reports indicate that activation of the kinase p38 MAPK might contribute significantly to the upregulation of PLA2s mRNAs and protein levels in mammalian cells [49].

The increased level of metabolites such as choline (Fig. 2c and 5) (1.40-fold, *P* = 0.0319 in sip63, 1.89-fold *P* < 0.001 in senescence) and GPC (Fig. 2c) (1.38-fold, *P* = n.s. in sip63; 1.64-fold *P* = < 0.014 in senescence) further confirmed an important enhancement in glycerophospholipid metabolism in both sets of cells.

Arachidonic acid (AA) is a peculiar PUFA produced from the sn2 position of glycerophospholipids after the action of PLA2. Arachidonic acid is the precursor of a family of more than 30 distinct lipid mediators with pro-inflammatory and/or homeostatic functions in the cell [50]. In our analysis, AA significantly doubled in both senescent (2.20-fold; *P* < 0.001) and sip63 (2.08-fold *P* = 0.0143) keratinocytes (Figs. 2b, c and 5), likely as a consequence of upregulation of *PLA2s* genes. Other free PUFAs that are likely released from the plasma membrane and upregulated in both sets of cells (Fig. 5) include eicosapentaenoic acid (1.85-fold, *P* < 0.001 in senescence and 1.54-fold, *P* = 0.0382 in sip63), docosapentaenoate (2.32-fold, *P* < 0.001 in senescence and 1.80-fold, *P* = 0.0138 in sip63). The synthesis of these highly unsaturated fatty acids plays an important role in the energetics of senescent cells, helping to recycle NAD+ for use in glycolysis when mitochondrial respiration is impaired, as occurs during various types of senescence [51]. In fact, we found that NAD+ levels were significantly upregulated in senescent keratinocytes (Fig. 5) (2.58-fold, *P* = 0.014). We do not have information on NADH levels and therefore on the NAD+/NADH ratio; however, analysis of nicotinamide phosphoribosyltransferase (NAMPT) expression (Fig. S3 and Table 1), the rate-limiting enzyme in the synthesis of NAD+ [52], did not reveal significative changes in mRNA levels in both p63-silenced and senescent keratinocytes, so NAD+ must originate from various NADH oxidizing reactions, or from its enhanced uptake, as is also referred to in “Discussion” section.

In summary, an altered metabolism of glycerophospholipids, also evidenced by the increase in its degradation products such as GPC and choline, is a common consequence of both the states of senescence and p63 silencing in human keratinocytes. Lyso-glycerophospholipids and free fatty acids originating from increased activity of phospholipases A1 and A2 could play crucial roles in cell signaling, inflammatory response and membrane remodeling in both conditions.

### Fatty acid metabolism is affected in senescence independently of p63

**Fig. 3:**
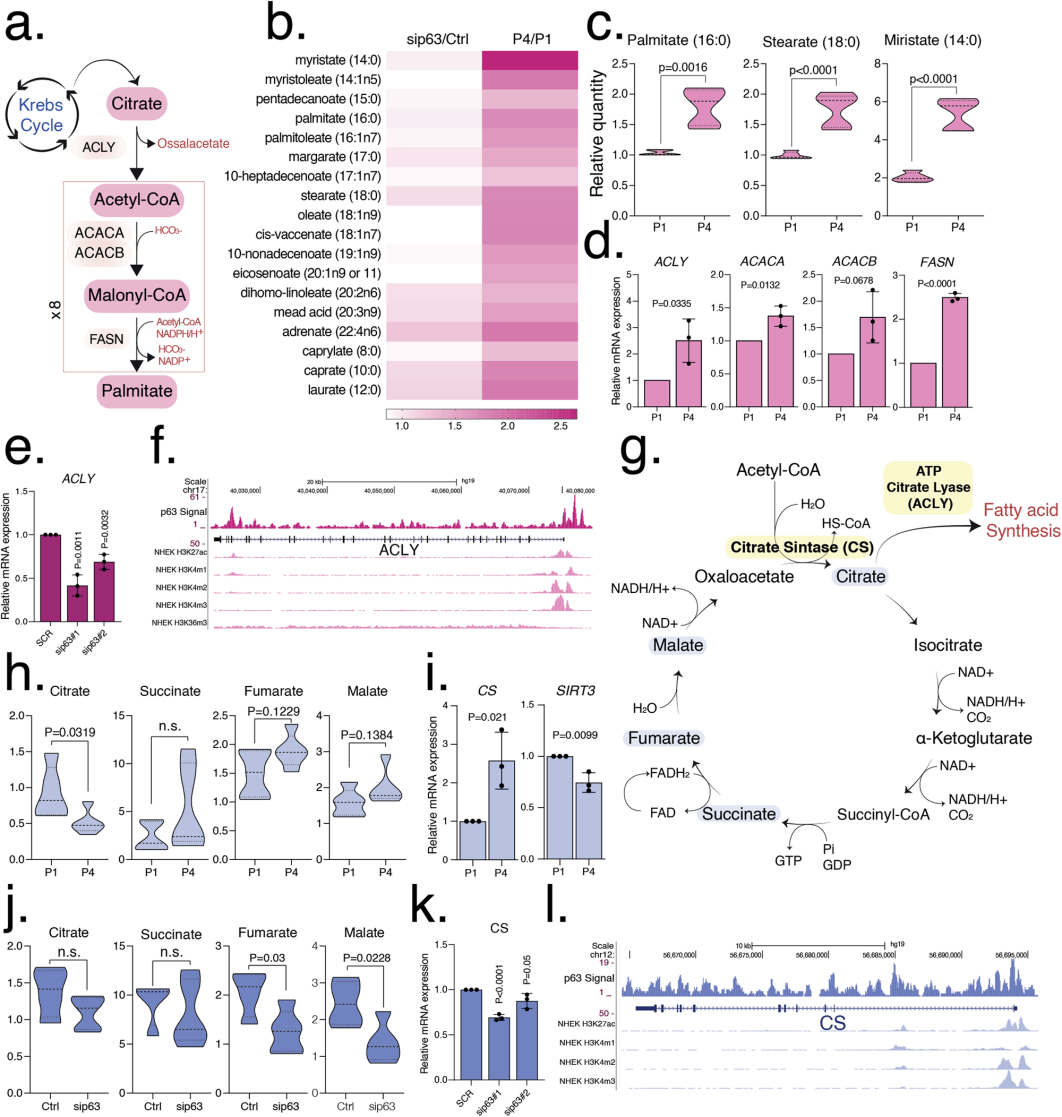
Increased fatty acid metabolism in senescent keratinocytes. **a** Graphical representation of the fatty acids synthesis pathway. **b** Heatmap shows the relative quantity of free fatty acids in senescent (P1, P4) and transfected (Ctrl, sip63 siRNAs) keratinocytes. Data are shown as mean of *N* = 5 biological replicates. **c** Relative quantity of palmitate (16:0), stearate (18:0) and myristate (14:0) in senescent (P1, P4) keratinocytes. Data are shown as mean ± SD of *N* = 5 biological replicates. The adjusted *p* values were calculated using Student’s *t*-test. **d** The mRNA expression levels of *ACLY, ACACA, ACACB* and *FASN* were evaluated using qRT-PCR in senescent (P1, P4) keratinocytes. Data are shown as mean ± SD of *N* = 3 biological replicates. The adjusted *p* values were calculated using Student’s *t*-test. **e** The mRNA expression level of *ACLY* was evaluated using qRT-PCR in transfected (Ctrl, sip63#1 and sip63#2 siRNAs) keratinocytes. Data are shown as mean ± SD of *N* = 3 biological replicates. The adjusted *p* values were calculated using Student’s *t*-test. **f** ChIP-seq signals profiles of p63, H3K27Ac, H3K4me1, H3K4me2, H3K4me3 and H3K36me3 (range 1–50) in NHEK at *ACLY* gene region. **g** Graphical representation of the Krebs cycle. **h** Relative quantity of citrate, succinate, fumarate and malate in senescent (P1, P4) keratinocytes. Data are shown as mean ± SD of *N* = 5 biological replicates. The adjusted *p* values were calculated using Student’s *t*-test. n.s. non-significant. **i** The mRNA expression levels of *CS* and *SIRT3* were evaluated using qRT-PCR in senescent (P1, P4) keratinocytes. Data are shown as mean ± SD of *N* = 3 biological replicates. The adjusted *p* values were calculated using Student’s *t*-test. **j** Relative quantity of citrate, succinate, fumarate and malate in transfected (Ctrl, sip63#1 and sip63#2 siRNAs) keratinocytes. Data are shown as mean ± SD of *N* = 5 biological replicates. The adjusted *p* values were calculated using Student’s *t*-test. n.s. non-significant. **k** The mRNA expression level of *CS* was evaluated using qRT-PCR in transfected (Ctrl, sip63#1 and sip63#2 siRNAs) keratinocytes. Data are shown as mean ± SD of *N* = 3 biological replicates. The adjusted *p* values were calculated using Student’s *t*-test. **l** ChIP-seq signals profiles of p63, H3K27Ac, H3K4me1, H3K4me2, H3K4me3 (range 1–50) in NHEK at *CS* gene region.

In senescence, our metabolomic analysis showed an intense upregulation of saturated free fatty acids (Fig. 5) such as palmitate (1.73-fold, *P* = 0.0016), stearate (1.76-fold, *P* < 0.0001), myristate (2.65-fold, *P* < 0.0001), and unsaturated free fatty acids including myristoleate (1.9-fold, *P* = 0.0159), mead acid (1.55-fold, *P* = 0.0538), dihomo-linoleate (1.38-fold, *P* = 0.0751), adrenic acid (1.91-fold, *P* = 0.0083). On the contrary, no variations were observed after silencing of p63 (Figs. 3a–c and 5). To check whether this increase in free fatty acids was subsequent to their enhanced biosynthesis, we investigated the mRNA levels of some enzymes relevant in fatty acid biosynthesis. In agreement with our metabolomic results, we found increased mRNA levels of fatty acid synthase (*FASN*) (2.49-fold, *P* < 0.0001), two isoforms of acetyl-CoA carboxylase (*ACACA*, *ACACB*) (1.37-fold, *P* = 0.032 and 1.69-fold, *P* = 0.0678, respectively) and citrate lyase (*ACLY*) (2.51 fold, *P* = 0.0335) which all increased significantly (or showed a trend towards a higher expression) in senescence (Fig. 3a, d and Table 1) but not in sip63 (Fig. S3 and Table 1). In contrast, *ACLY* (0.42-fold, *P* = 0.0011), was significantly downregulated in p63 silencing (Fig. 3e and Table 1), confirming that p63 does not contribute to increase fatty acid synthesis in human keratinocytes. In the latter case, a clear p63 ChIP signal is detected in the promoter of the *ACLY* gene in a region of open chromatin state (GSE59827), suggesting the possibility that p63 could directly positively regulate the *ACLY* gene expression (Fig. 3f). This dependence of ACLY expression from ΔNp63 has also been previously reported in colon cancer stem cells [53]. Of note, mRNA levels of two major regulators of lipid synthesis SREBP1 and SREBP2 were not affected in senescent keratinocytes or in p63-silenced keratinocytes (Fig. S3). Similarly, we noticed a senescence-restricted increase in cholesterol (1.43-fold; *P* = 0.0646) and cortisol (1.49-fold, *P* = 0.0047) (Fig. 5). In senescent cells, these two compounds can be produced or most likely absorbed from the medium. Of note, this was not observed in sip63 cells. Altogether, in senescent keratinocytes, free fatty acids, enzymes required for lipid synthesis and cholesterol are prominently and significantly upregulated, but not in p63-silenced cells.

### p63 controls Krebs cycle

The main source of carbon atom in fatty acid synthesis is acetyl-CoA derived from Krebs cycle citrate [54]. Therefore, we checked Krebs cycle metabolites in our senescence analysis (Figs. 3g, h and 5) and found a significant decrease in citrate (0.56-fold, *P* = 0.0319), but an increase in mRNA level for citrate synthase (*CS*) (2.58-fold, *P* = 0.021) (Fig. 3i and Table 1). These two latter findings suggest an improved utilization of citrate in senescent keratinocytes that is probably continuously exported in the cytoplasm for fatty acid synthesis. The flow of metabolites through the Krebs cycle is also allosterically regulated. In this regard, in senescent cells, feedback inhibition of citrate synthase due to excess citrate could be alleviated by its export into the cytosol. Furthermore, the availability of one of its substrates, acetyl-CoA, could be reduced by inhibitory phosphorylation of the pyruvate dehydrogenase complex (PDH) as described in the next paragraph [54]. Succinate, fumarate and malate showed a consistent trend between senescence and controls, with no significant variations (Figs. 3h and 5). Senescent cells were characterized by high levels of NAD+ (2.58-fold, *P* = 0.0140) (Fig. 5). One potential source of NAD+ is the reversible reactions of the cytosolic malate dehydrogenase MDH1 and its mitochondrial isoenzyme MDH2. In the last step of the Krebs cycle, MDH2 is involved in the recycling of oxaloacetate from malate with the production of one molecule of NADH. Instead, MDH1 is mainly responsible for the opposite intermembrane reaction, which is the reduction of oxaloacetate to malate with the production of NAD+ and, together with MDH2 participates in the malate-aspartate shuttle whose function is to transport reducing NADH equivalents from cytosol to mitochondria [55]. We then questioned mRNA levels of *MDH1* and *MDH2* in senescent keratinocytes; however, we found that the levels of both isoenzymes were not significantly changed (Fig. S3 and Table 1). Therefore, due to the reversibility of both MDH1 and MDH2 reactions and the consistent levels of malate between senescent cells and controls, it is not possible to ascribe the high levels of NAD+ detected in senescent keratinocytes to the increased activity of MDH isoenzymes.

p63 controls aerobic metabolism [22], so in its absence we expected a decrease in the levels of the metabolites downstream of glycolysis, such as those in the Krebs cycle. Indeed, in our results, Krebs metabolites such as fumarate (0.61-fold, *P* = 0.0373) and malate (0.56-fold, *P* = 0.0228) were significantly downregulated or show a downregulating trend (citrate or succinate). In sip63, *MDH1* and *MDH2* mRNA levels show consistent values compared to controls (Fig. S3 and Table 1) while *CS* mRNA levels were slightly downregulated (0.69 fold, *P* = < 0.0001) (Figs. 3k and 5). Thus, together with *ACLY*, *CS* might be positively regulated by p63 although p63 occupancy at *CS* locus was weak in p63 ChIP-seq data (GSE59827) and more experiments are needed to prove the direct link between CS and p63 (Fig. 3l and Table 1). In summary, loss of p63 leads to a decrease in levels of Krebs cycle metabolites, along with reduced levels of CS. On the contrary, CS is increased during senescence.

### p63 and induction of senescence have distinct effects on glycolysis

**Fig. 4:**
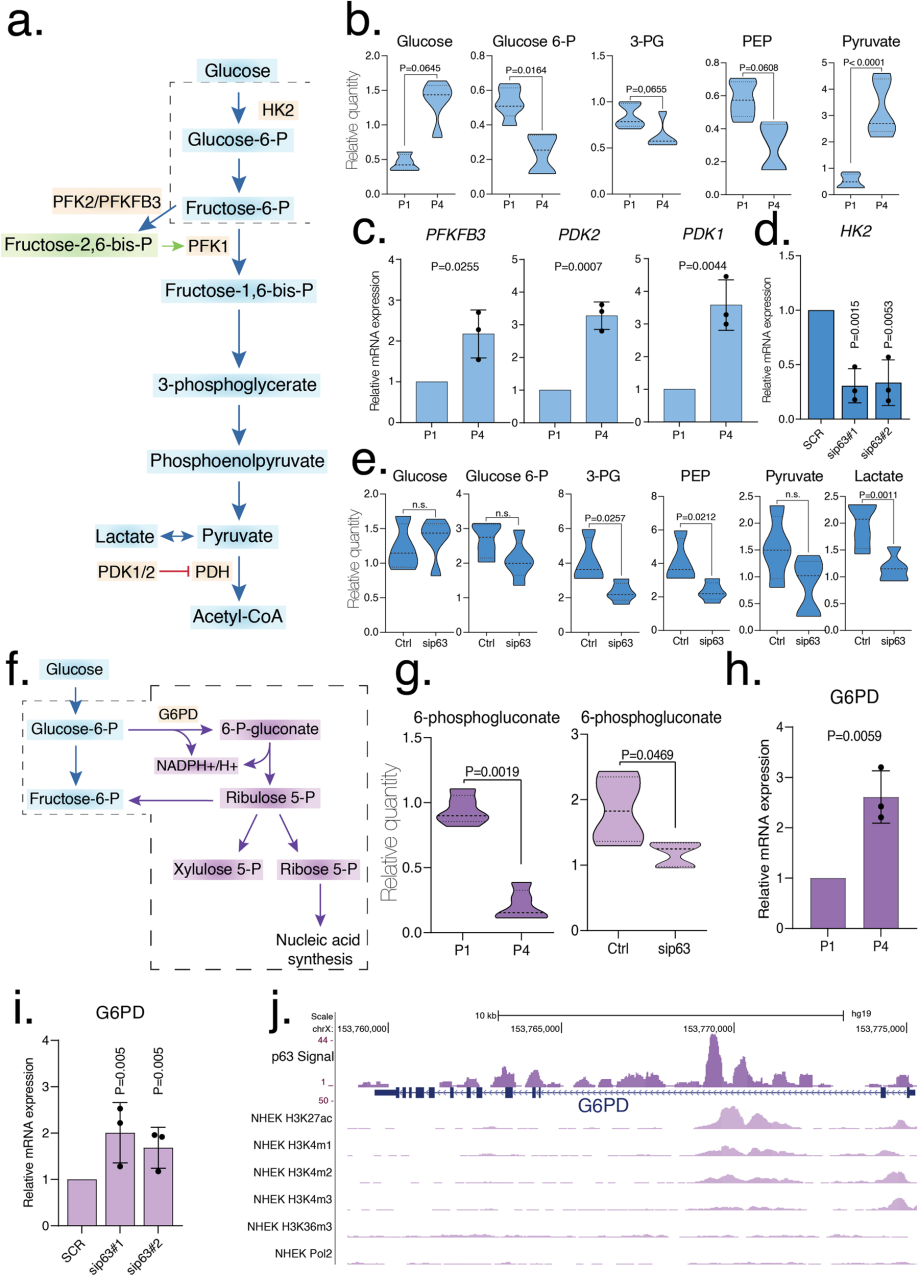
Altered glucose metabolism. **a** Graphical representation of glycolysis pathway. **b** Relative quantity of glucose, glucose-6-P, 3-PG, PEP and pyruvate in senescent (P1, P4) keratinocytes. Data are shown as mean ± SD of *N* = 5 biological replicates. The adjusted *p* values were calculated using Student’s *t*-test. **c** The mRNA expression levels of *PFKFB3, PDK2* and *PDK1* were evaluated using qRT-PCR in senescent (P1, P4) keratinocytes. Data are shown as mean ± SD of *N* = 3 biological replicates. The adjusted *p* values were calculated using Student’s *t*-test. **d** The mRNA expression level of *HK2* was evaluated using qRT-PCR in transfected (Ctrl, sip63#1 and sip63#2 siRNAs) keratinocytes. Data are shown as mean ± SD of *N* = 3 biological replicates. The adjusted *p* values were calculated using Student’s *t*-test. **e** Relative quantity of Glucose, Glucose-6-P, 3-PG, PEP, pyruvate and lactate in transfected (Ctrl, sip63 siRNAs) keratinocytes. Data are shown as mean ± SD of *N* = 5 biological replicates. The adjusted *p* values were calculated using Student’s *t*-test. n.s. non-significant. **f** Graphical representation of pentose phosphate pathway. **g** Relative quantity of 6-phosphogluconate in senescent (P1, P4) and transfected (Ctrl, sip63 siRNAs) keratinocytes. Data are shown as mean ± SD of *N* = 5 biological replicates. The adjusted *p* values were calculated using Student’s *t*-test. **h** The mRNA expression level of *G6PD* was evaluated using qRT-PCR in senescent (P1, P4) keratinocytes. Data are shown as mean ± SD of *N* = 3 biological replicates. The adjusted *p* values were calculated using Student’s *t*-test. **i** The mRNA expression level of *G6PD* was evaluated using qRT-PCR in transfected (Ctrl, sip63#1 and sip63#2 siRNAs) keratinocytes. Data are shown as mean ± SD of *N* = 3 biological replicates. The adjusted *p* values were calculated using Student’s *t*-test. **j** ChIP-seq signals profiles of p63, H3K27Ac, H3K4me1, H3K4me2, H3K4me3, H3K36me3 and Polymerase II (range 1–50) in NHEK at *G6PD* gene region.

**Fig. 5:**
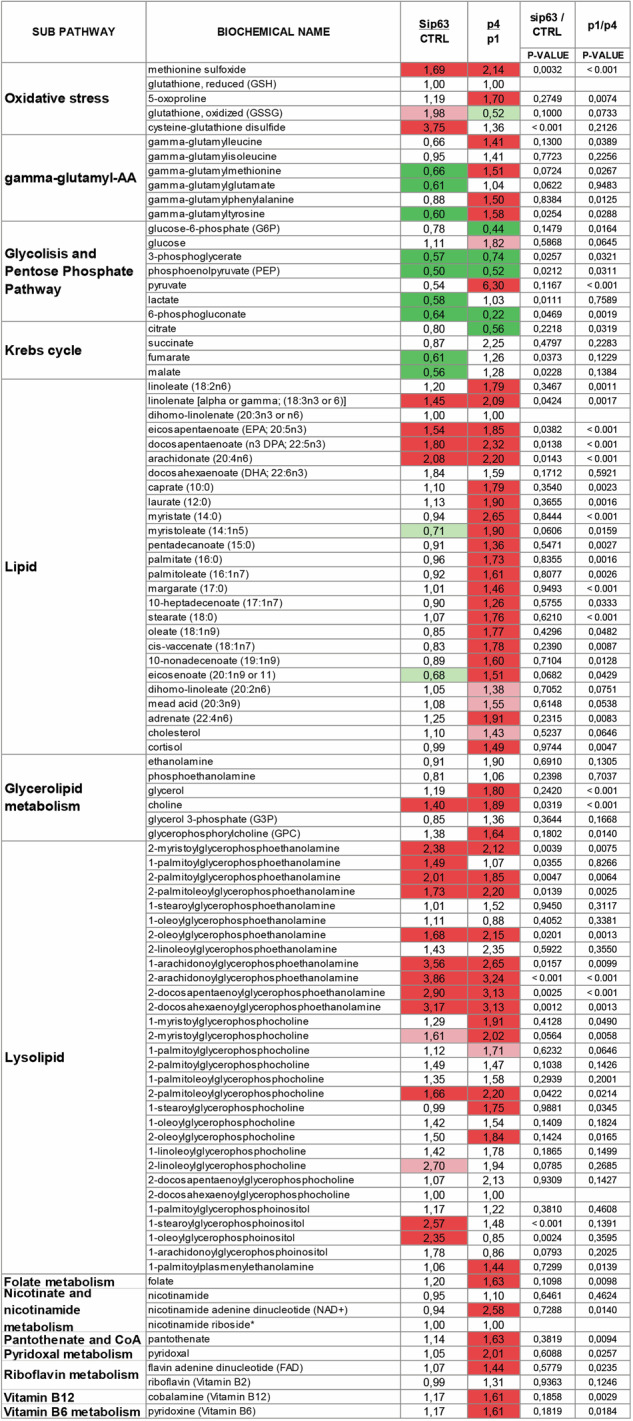
Summary of results from the metabolomic study. The pathway heatmap shows the relative ratios, or fold of change values, between biochemicals detected in the sip63 keratinocytes vs control, and P4 senescent vs P1 control keratinocytes. Green and coral shading indicate statistically significant (*P* ≤ 0.05) decreases or increases in fold of change, respectively. Light green and light pink shading indicate a trend (0.1 ≥ *P* ≥ 0.05).

In senescence, glycolytic metabolites such as glucose (1.82-fold, *P* = 0.0645) and mainly pyruvate (6.30-fold; *P* < 0.001) are both upregulated, while glucose-6-phosphate (glucose-6-P, 0.44-fold, *P* = 0.0164), 3-phosphoglycerate (3-PG, 0.74-fold, *P* = 0.0321) and phosphoenolpyruvate (PEP, 0.52-fold, *P* = 0.311) are significantly downregulated (Figs. 4a, b and 5). The senescence state probably fosters glucose uptake [56]. To uncover the origin of the increase of pyruvate in P4 cells, we investigated the mRNA levels of two isoforms of pyruvate kinase *PKM1* and *PKM2* in P4 keratinocytes and found marked upregulating trends in both enzymes in P4 compared to P1 (Fig. S5b and Table 1), although they did not reach statistical significance. PKM2 is also under tight allosteric control by F16BP (fructose-1,6-bisphosphate) which favors its tetrameric active state and reduce cell proliferation in HCT-116 cells [57]. Other possibilities to regulate PKM2 activity include post-translational modification such as acetylation, phosphorylation and proline hydroxylation that are not considered in this study [57]. We then decided to gain insights into reactions that utilize pyruvate downstream of glycolysis, such as the PDH which converts pyruvate into acetyl-CoA. PDH subunit E_1_ is mainly regulated by phosphorylation by one of the four inactivating kinases, named PDK1-4 [58]. Both *PDK1* (3.58-fold, *P* = 0.0044) and *PDK2* (3.28-fold, *P* = 0.0007) mRNA level showed significative upregulation in senescent keratinocytes compared to control, while *PDK3* and *PDK4* were not detected (Fig. 4c; Fig. S5 and Table 1). Thus, PDKs increase activity probably slows down the action of PDH with consequent pyruvate accumulation.

Moreover, allosteric inhibition of PDH by long-chain fatty acids, upregulated in senescent keratinocytes compared to controls, could contribute to reduced PDH activity [54]. We do not know if pyruvate is involved in lactic fermentation. Indeed, although intracellular lactate levels are consistent between P1 and P4 keratinocytes (Fig. 5), we are not aware of its extracellular concentration, as lactate is usually excreted. To evaluate changes in transcription of the other two enzymes involved in the control of glycolytic flux, we evaluated mRNA level of hexokinase 2 (HK2) and PFKFB3, the isoform of 6-phosphofructo-2-kinase/fructose-2,6-biphosphatase [59] with the highest kinase:phosphatase ratio, which synthetize fructose-2,6-bisphosphate (F2,6P2) the most powerful allosteric regulator of phosphofructokinase-1 (PFK-1), which in turn sustains glycolysis. The level of *HK2* mRNA was comparable with controls (Fig. S5b and Table 1), while the level of *PFKFB3* mRNA (Fig. 4c and Table 1) was significantly increased in senescence (2.18-fold, *P* = 0.0255), in agreement with increased pyruvate levels.

In keratinocytes, p63 positively controls HK2 [22], which as expected showed a drastic downregulation upon p63 silencing (0.30-fold, *P* = 0.0015) (Fig. 4d and Table 1). Therefore, in sip63 keratinocytes, glycolytic metabolites downstream of glucose such as 3-PG (0.57-fold, *P* = 0.0257) and PEP (0.50-fold, *P* = 0.0212) show a significant downregulation, or a downregulating trend such as glucose-6-P and pyruvate (Figs. 4a, e and 5). The mRNA levels of other enzymes that control glycolytic flow, namely *PFKFB3*, *PMK1* and *PMK2* were similar to the controls in sip63 (Fig. S5a and Table 1). To sum up, depletion of p63 leads to decrease of glycolysis, which is otherwise activated in senescent cells, probably via upregulation of PFKB3 and improved uptake of glucose from the media.

### 6-phosphogluconate is reduced in senescent cells in p63-dependent manner

In the PPP, our targeted metabolomic analysis included only 6-phosphogluconate (Figs. 4f, g and 5), which is the product of the first oxidation reaction and which was found to be decreased in sip63 (0.64-fold, *P* = 0.0469) with a more pronounced effect (0.22-fold, *P* = 0.0019) in senescent keratinocytes. We then investigated the mRNA levels of the corresponding enzyme glucose-6-phosphate dehydrogenase (*G6PD*), the first of the pathway and also the regulatory, and found that in both sets of samples, *G6PD* mRNA levels were significantly upregulated (2.01-fold, *P* = 0.005 in sip63 and 2.26-fold, *P* = 0.0059 in senescence) (Figs. 4h, i and 5). Furthermore, analysis of the p63 ChIP-seq data in proliferating keratinocytes revealed a possible p63 binding site in the second intron of the *G6PD* gene (Fig. 4j) in a region of open chromatin (GSE59827). However, it must be considered that post-translational modifications such as O-GlcNAcylation, phosphorylation and lysine acetylation at multiple sites can also modulate G6PD activity [60]. No differences were detected in the levels of 6-phosphogluconate dehydrogenase (*PGD*) mRNA level, the enzyme responsible for the second, not limiting, oxidative step of PPP in both conditions (Fig. S5b and Table 1). Altogether upregulation of the G6PD and phosphogluconate significant reduction lead us to speculate that PPP pathway is tuned up in both sets of cells to fulfill the continuous requirement for NADPH-reducing power as a response to the increased oxidative stress.

### *SIRT3* is downregulated during senescence

Sirtuins are a family of deacetylating enzymes that are involved in the regulation of various cellular processes such as metabolism, stress response, genome instability and aging [61]. This led us to study the mRNA expression of the seven human sirtuins, to evaluate their possible role in the metabolic changes of sip63 and senescent keratinocytes. We found that *SIRT2* and *SIRT4* mRNAs were not detected in NHEKs, while *SIRT1*, *SIRT5*, *SIRT6* and *SIRT7* mRNA levels in both senescence and in sip63 were comparable with controls (Fig. S4a, b and Table 1). Of note, the expression of mitochondrial *SIRT3* was instead significantly reduced in senescent cells (0.74-fold, *P* = 0.0099) (Fig. 3i and Table 1) but not in sip63 keratinocytes (Fig. S4a and Table 1). This finding is in agreement with previous reports suggesting that SIRT3 depletion is partially responsible for the senescence program [62].

### Vitamins and cofactor levels vary in senescent keratinocytes

Senescent cells are known to be metabolically active, although in a growth-arrested state. This is confirmed by an increase in intracellular concentration of vitamins and cofactors (Fig. 5) such as folate (1.63-fold, *P* = 0,0098), pantothenate (1.63-fold, *P* = 0,0094), pyridoxal (2.01-fold, *P* = 0.0257), pyridoxine (1.61-fold, *P* = 0.0184), cobalamin (1.61-fold, *P* = 0.0029), flavin adenine dinucleotide (FAD) (1.44-fold, *P* = 0.0235) and NAD+ (2.58-fold, *P* = 0.0140). We believe that such a significant increase in vitamins and/or cofactors could be due to enhanced uptake from the media, possibly promoted by the senescence phenotype. No major changes in the above metabolite were found in sip63 keratinocytes suggesting a p63-independent regulation (Fig. 5).

## Discussion

Redox balance, involving glutathionyl conjugates, thioredoxin system or glutathione *S*-transferase are essential to regulate biological cell processes, including cell fate [63–65]. Indeed, gas-transmitter and the Fenton reaction are at the heart of ferroptosis [66, 67], regulating crucial metabolic patterns in normal as well as cancer cells [68–70]. Here, we dissected the metabolic pathways associated in senescent or p63-depleted keratinocytes to identify the p63-governed metabolic pathways that may be responsible for the senescence phenotype. Our study is based on steady-state levels of metabolites and gene expression analysis of enzymes involved in relevant biochemical pathways. We detected important alterations in the metabolomic profiles of oxidative stress markers, likely derived from the imbalance of the ROS production/neutralization ratio in both senescent and sip63 cells. DNA damage, telomere damage, and the activation of oncogenes can trigger events that, through pathways that are still mechanistically unclear, foster the production of ROS from mitochondrial and non-mitochondrial sources [33]. On the other hand, p63 protects cells from oxidative stress by controlling genes involved in glutathione metabolism that antagonize ROS [43], or by activating ROS scavenger genes such as cytoglobin [25]. Our work suggests that loss of p63 during senescence can lead to increasing levels of methionine sulfoxide via enhanced ROS production. In fact, methionine actively participates in the maintenance of cellular redox homeostasis and protects proteins from irreversible oxidative damage [34, 71, 72]. Being also a methyl donor and an essential component of proteins, methionine oxidation can have a profound impact on the structure and function of proteins and consequently on cellular metabolic homeostasis and organismal aging [72]. Of note, expression of several enzymes, including glutathione recycling (GGT5), glutathione reduction (GSR) and ROS detoxification (GSTP1) genes, involved in ROS pathway was downregulated in the absence of p63 yet unchanged or slightly increased in senescent keratinocytes, suggesting only a partial impact of p63 loss on senescent induction via ROS accumulation. The undetectable value of reduced glutathione (GSH), under normal or altered conditions, in both sets of cells probably reflects a general over activity of GGT enzymes in the outer membrane of keratinocytes where reduced glutathione is recycled and regenerated at a high rate. If this holds, GGT activity could drain GSH away from the local environment and negatively impact the intracellular GSH/GSSG redox ratio in keratinocytes [36]. However, it must also be considered that the GSH/GSSG ratio in keratinocytes is extremely low as has previously been demonstrated compared to fibroblasts [73], or after UV exposure [74, 75]. Indeed, increased levels of 5-oxoproline and gamma-glutamyl amino acids (Fig. 5) suggest an improved gamma-glutamyl cycle in senescent keratinocytes. On the other hand, enhanced expression of OPLAH protects sip63 keratinocytes from excess of 5-oxoprolin, which is also a marker of oxidative stress. Cysteine glutathione disulfide is the second oxidized sulfur-containing amino acid that is upregulated in p63-silenced cells and confirms the need for keratinocytes to activate alternative cellular defense against increased oxidative stress caused by p63 depletion [35]. It is thus plausible that the strong antioxidant activity of p63, in addition to protecting keratinocytes in the proliferative compartment of the epidermis, can also inhibit their differentiation. In the suprabasal layers of the epidermis, low levels of ΔNp63 and consequent ROS accumulation might then unleash keratinocyte differentiation [73].

Our data suggest that p63 inhibits the production of lyso-glycerophospholipids and arachidonic acid. These bioactive signaling lipid molecules resulting from the hydrolytic action of PLA1 and PLA2 on the position sn1 and sn2 of glycerophospholipids, act as important mediators of senescence and/or inflammatory signatures [76], and are both upregulated in senescent and sip63 keratinocytes. In particular, two LPE lysophospholipids, namely 1-arachidonoyl-GPE and 2-arachidonoyl-GPE, are increased in senescent cells possibly due to p63 loss. These two molecules, once oxidized, generate a novel family of signaling metabolites called eicosanoid–lysolipids and recent reports suggest their involvement in inflammation and aging pathways [77, 78]. Mechanistically, we suggest that p63 binds to genomic loci of a family of PLA genes to repress their transcription. Accordingly, we also observed an increase of arachidonic acid, a product of PLA activity, in p63-depleted or senescent cells, reinforcing the importance of this pathway in senescence. Our screen of mRNA levels of the seven sirtuins did not reveal important modulations except for a decrease of *SIRT3* expression in senescence. However, because of the important role that these proteins play in the regulation of metabolism, we cannot exclude their variation at the protein level in both sets of cells, as it has already been proved for SIRT1 in senescent keratinocytes [21].

Fatty acid metabolism was reported to be important during senescence to promote and maintain high fatty acid cell content [79, 80]. In agreement with these data, we observed an extensive upregulation of fatty acid species both saturated and unsaturated, and the concomitant improved expression of the enzymes necessary to their synthesis, such as FASN, ACACA, ACACB and ACLY in senescent keratinocytes. Despite previous work indicating a possible regulation of *FASN* by p63 [26, 81–83], in our model, we did not observe any significant changes of *FASN*, *ACACA* and *ACAC*B expression nor in in fatty acids levels upon p63 knockdown suggesting that upregulation of this pathway in senescence is p63-independent. In human keratinocytes, p63 supports aerobic respiration through positive transcriptional control of HK2, one of the main booster of the glycolytic pathway [22]. As expected, in p63-silenced keratinocytes glycolytic metabolites are significantly decreased or have a downregulating trend. Furthermore, as a direct consequence of the key role played by the p63-HK2 axis in supporting mitochondrial oxidative metabolism, a decrease in Krebs cycle metabolites and its probable impairment were observed after silencing of p63. These changes might also be explained by a possible direct regulation of CS (citrate synthase) by p63. The replicative senescence of cultured cells is characterized by increased glucose uptake and a metabolic shift toward glycolytic metabolism and lactate production [84, 85]. Similarly, our senescent keratinocytes appear to adopt an increased glycolytic state, as evidenced by a significant and intense accumulation of its end product, namely pyruvate. Although the major source of pyruvate is glycolysis, it can also be produced from alanine or lactate. However, no alterations of alanine (data not shown) or lactate levels were detected. In fact, increased mRNA levels of key glycolysis-promoting enzymes such as PFKFB3, PKM1 and PKM2 appear to sustain the glycolytic origin of pyruvate. Moreover, pyruvate build-up can also be attributed to decreased activity of the mitochondrial gatekeeper PDH complex, due to inhibitory phosphorylation by two kinases PDK1 and PDK2 that we found significantly upregulated in senescence [58]. We are tempted to hypothesize that an increase in pyruvate can be a late response of the keratinocyte to the senescence program, which is indeed a dynamic process [86]. Pyruvate protects cells from increased oxidative stress by scavenging ROS [87]. Malic enzyme (ME) activity may also be a source of pyruvate, but although malate levels are unchanged, the contribution of ME to pyruvate level during keratinocyte senescence cannot be excluded [88]. The increased glucose level and concomitant decrease in glucose-6-phosphate likely reflect the need for senescent cells to boost up glucose uptake which, once phosphorylated [56], can fuel both glycolysis and the PPP. In fact, the latter is the main source of NADPH crucial for glutathione reduction and ROS detoxification in both sip63 and senescent cells. We hypothesize that the rapid utilization and removal of products such as NADPH from the pentose phosphate shunt increases the flux of metabolites through the pathway. This is also evidenced in both sets of cells by elevated levels of G6PD, the first and rate-limiting enzyme of the PPP, whose expression is potentially regulated by p63. Furthermore, senescent keratinocytes can utilize NADPH also as an anabolic reducing power for the synthesis of fatty acids. Accordingly, a decline of citrate levels and the increase in the levels of CS and ACLY are consistent with amplified utilization of citrate for the synthesis of fatty acids in the enlarged and flattened senescent cells [89]. Low levels of citrate also agree with increased glycolysis flux and pyruvate production, since citrate has inhibitory action on PFK [54].

In senescence, the significant increase in NAD+ levels, which is necessary for use in glycolysis, does not appear to be attributable to reversible reactions of MDH1 and MDH2, since both enzymes do not increase significantly and since malate levels are consistent between senescence and controls. Furthermore, the possibility of de novo NAD+ biosynthesis is excluded by consistent levels of NAMPT in senescent keratinocyte vs controls. Rather, NAD+ in senescence may be potentially produced during unsaturation of fatty acids or lactate production, the latter usually excreted and thus not detectable in our analysis as described before. It has long been known that some forms of senescence are caused by mitochondrial dysfunction (MiDAS) [62], characterized by a lowering of the NAD+/NADH ratio, data that, however, are not present among our available metabolomics compounds. Furthermore, the present study does not include an analysis of mitochondrial morphology and function, which will certainly add important insights into energetics, ATP production, and NAD+ recycling in both senescent and sip63 keratinocytes. Of course, we do not exclude that the accumulation of NAD+ may be the result of increased uptake from the culture medium as observed for other vitamins or cofactors. Indeed, elevated levels of folate, pantothenate, pyridoxal, pyridoxine, FAD and cobalamin detected are probably required for the maintenance of the highly demanding senescent phenotype in keratinocytes where they can also help in counteracting oxidative stress and inflammation. Thus, while glycolysis and PPP in sip63 seems to have opposite trends, in senescent keratinocytes, our data suggest an increase in glucose-6-phosphate flux through both glycolysis and the PPP resulting in a greater production of pyruvate, and perhaps also NADPH-reducing power and lactate, although we do not have a direct evidence for changes in the latter two metabolites.

In conclusion, here we show that markers of oxidative stress, lyso-glycerophospholipid metabolism and PPP have a similar metabolic profile in both senescent and p63-silenced keratinocytes probably because both conditions share increased oxidant species, inflammation state and high demand of NADPH-reducing power to antagonize ROS production. Our data also uncovered the enhanced action of PLA1/2, probably under the control of p63, which generates crucial bioactive lipid species whose intriguing role in provoking and sustaining the state of senescence and/or inflammation certainly deserves further investigation. Our targeted metabolomic analyses did not allow us to detect and evaluate the level of other metabolites that could have been useful in integrating our study. In addition, although we have assessed the expression of several relevant metabolic enzymes, we have no information on either the corresponding protein level or their regulation through post-translational modifications or the concentration of their allosteric effectors and inhibitors. Our data indicate that p63 might contribute to the establishment of senescence also through its impact on cellular metabolism; however, at the moment causative relationships are missing. Further work is needed to address these limitations, for instance, analysis of mitochondrial morphology, energetics and physiology of senescent keratinocytes, which would add an interesting functional frame to the biochemical and transcriptional picture here described. However, we believe that our omics analyses unveiled important metabolic links between p63 and keratinocyte senescence.

## Materials and methods

### Experimental design

Global biochemical profiles were compared across the following sample groups (biological replicates):

- CTRL, siRNA controls (*n* = 4);
- Sip63, p63 silenced (*n* = 5);
- p1 young keratinocytes, control (*n* = 5);
- p4 senescent keratinocytes (*n* = 5).

### Cell culture and transfection

Primary normal human epidermal keratinocytes (NHEK) (Gibco, catalog no. C-001-5C) cultured in EpiLife medium with the addition of Human Keratinocyte Growth Supplements (HKGS, Life Technologies). Cells were grown at 37 °C and 5% CO_2_ in a humidified atmosphere and kept constantly sub-confluent to avoid triggering of differentiation. At each passage, cells were harvested, counted and seeded to calculate the population doublings and the population doubling time. At each passage, cells were collected to extract RNA and proteins, and an aliquot was submitted to senescence-activated (SA) β-galactosidase staining. For p63 siRNA-mediated knockdown experiments, 3 × 10^5^ cells were seeded and transfected with specific siRNAs (p63-1 UGAAUUCAGUGCCAACCUG; p63-2 CAGGUUGGCACUGAAUUCA; siSCR CAGAGAGAACUCAAACGCCAAUGCU) using Lipofectamine RNAiMAX transfection reagent (Invitrogen). The knockdown efficiency of p63 was evaluated by western blotting or qPCR analysis 48 h after transfection.

### Metabolon summary of procedure

Metabolic analysis was performed by Metabolon, Inc. (Morrisville, NC 27560, USA) on 19 samples of neonatal human keratinocytes (NHEK). As indicated by mView™ REPORT provided by Metabolon Inc., following receipt, samples were inventoried, and immediately stored at −80 °C. At the time of analysis, samples were extracted and prepared for analysis using Metabolon’s standard solvent extraction method. The extracted samples were split into equal parts for analysis on the GC/MS and LC/MS/MS platforms. Also included were several technical replicate samples created from a homogeneous pool containing a small amount of all study samples (“Client Matrix”) [90–93]. Each sample received was accessioned into the Metabolon LIMS system and was assigned by the LIMS a unique identifier, which was associated with the original source identifier only. This identifier was used to track all sample handling, tasks, results, etc. The samples (and all derived aliquots) were bar-coded and tracked by the LIMS system. All portions of any sample were automatically assigned their own unique identifiers by the LIMS when a new task was created; the relationship of these samples was also tracked. All samples were maintained at −80 °C until processed. The sample preparation process was carried out using the automated MicroLab STAR® system from Hamilton Company. Recovery standards were added prior to the first step in the extraction process for QC purposes. Sample preparation was conducted using a proprietary series of organic and aqueous extractions to remove the protein fraction while allowing maximum recovery of small molecules. The resulting extract was divided into two fractions; one for analysis by LC and one for analysis by GC. Samples were placed briefly on a TurboVap® (Zymark) to remove the organic solvent. Each sample was then frozen and dried under vacuum. Samples were then prepared for the appropriate instrument, either LC/MS or GC/MS.

#### Liquid chromatography/mass spectrometry (LC/MS, LC/MS^2^)

The LC/MS portion of the platform was based on a Waters ACQUITY UPLC and a Thermo-Finnigan LTQ mass spectrometer, which consisted of an electrospray ionization source and linear ion-trap (LIT) mass analyzer. The sample extract was split into two aliquots, dried, then reconstituted in acidic or basic LC-compatible solvents, each of which contained 11 or more injection standards at fixed concentrations. One aliquot was analyzed using acidic positive ion optimized conditions and the other using basic negative ion optimized conditions in two independent injections using separate dedicated columns. Extracts reconstituted in acidic conditions were gradient eluted using water and methanol both containing 0.1% formic acid, while the basic extracts, which also used water/methanol, contained 6.5 mM ammonium bicarbonate. The MS analysis alternated between MS and data-dependent MS^2^ scans using dynamic exclusion.

#### Gas chromatography/mass spectrometry (GC/MS)

The samples destined for GC/MS analysis were re-dried under vacuum desiccation for a minimum of 24 h prior to being derivatized under dried nitrogen using bistrimethyl-silyl-trifluoroacetamide (BSTFA). The GC column was 5% phenyl and the temperature ramp is from 40° to 300 °C in a 16 min period. Samples were analyzed on a Thermo-Finnigan Trace DSQ fast-scanning single-quadrupole mass spectrometer using electron impact ionization. The instrument was tuned and calibrated for mass resolution and mass accuracy on a daily basis. The information output from the raw data files was automatically extracted as discussed below.

#### Accurate mass determination and MS/MS fragmentation (LC/MS), (LC/MS/MS)

The LC/MS portion of the platform was based on a Waters ACQUITY UPLC and a Thermo-Finnigan LTQ-FT mass spectrometer, which had a LIT front end and a Fourier transform ion cyclotron resonance mass spectrometer backend. For ions with counts >2 million, an accurate mass measurement could be performed. Accurate mass measurements could be made on the parent ion as well as fragments. The typical mass error was <5 ppm. Ions with <2 million counts require a greater amount of effort to characterize. Fragmentation spectra (MS/MS) were typically generated in data-dependent manner, but if necessary, targeted MS/MS could be employed, such as in the case of lower level signals. Additional information regarding Metabolon analysis of keratinocytes samples is referred in the Supplementary Material.

### RNA extraction and RT-qPCR

HEKn cells were lysed in RNeasy Lysis Buffer (RLT) (QIAGEN). Total RNA was isolated using the RNeasy Mini Kit (QIAGEN). Total RNA was quantified using a NanoDrop spectrophotometer (Thermo Fisher Scientific). Total RNA was used for complementary DNA (cDNA) synthesis with a SensiFAST cDNA synthesis kit (Bioline). Real-time qPCR was performed with the PowerUp SYBER Green Master Mix (Applied Biosystem), using appropriate qPCR primers (listed in Table S1). TATA box-binding protein was used as a housekeeping gene for data normalization. The expression of each gene was defined by the threshold cycle (Ct), and relative expression levels were calculated by using the 2^−ΔΔCt^ method. Statistical analysis was performed using GraphPad Prism 9.5.1.733.

### Western blotting

Total cell extracts were resolved on a SDS polyacrylamide gel and blotted on Amersham Hybond PVDF membranes (GE Healthcare). Membranes were incubated with primary antibodies overnight at 4 °C, washed and hybridized for 1 h at room temperature using the appropriate secondary antibody (rabbit and mouse; Bio-Rad, Hercules, CA, USA). The following primary antibodies were used: anti-p63 (Cell Signaling, D9L7L); anti-p16 (Santa Cruz Biotechnology (JC8): sc-56330), anti-p53 (Santa Cruz Biotechnology (DO-1): sc-126), anti-β-actin (Sigma, AC-15: A5441) and anti-GAPDH (Sigma: G8795). The uncropped blot files are in the Supplementary Material.

## Supplementary information

Supplemental Material
