# Supplementary material for: p63 affects distinct metabolic pathways during keratinocyte senescence, evaluated by metabolomic profile and gene expression analysis
Source: Cell Death Dis. 2024 Nov 14;15(11):830. doi: 10.1038/s41419-024-07159-7 (PMC11564703; doi:10.1038/s41419-024-07159-7)
Supplement: Supplementary file 1 — Supplemental Material [file 41419_2024_7159_MOESM1_ESM.pdf]

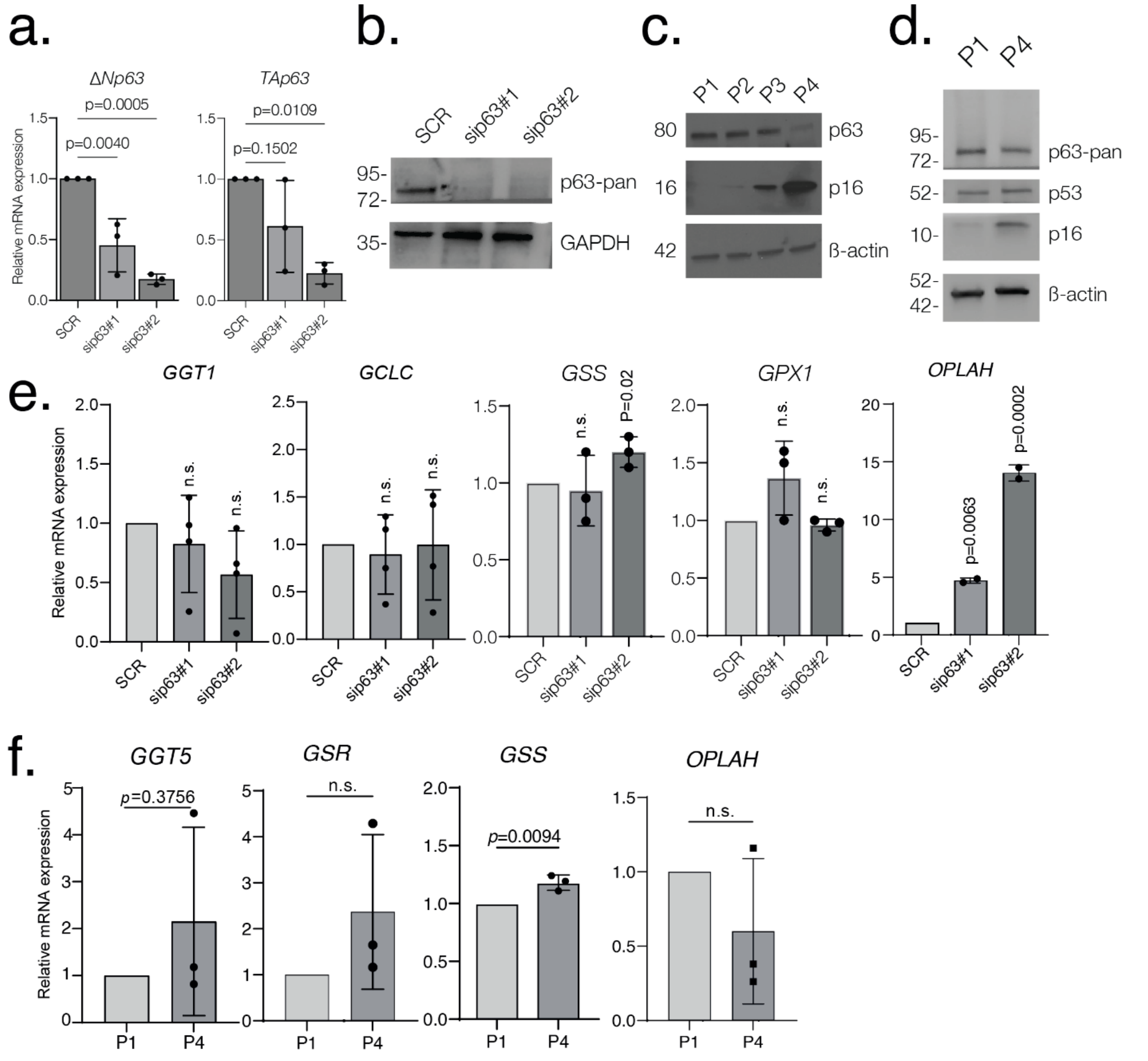

Fig. S1. Increased oxidative stress. A) The mRNA expression level of ΔNp63 and TAp63 were evaluated using qRT-PCR in transfected (Ctrl, sip63#1 and sip63#2 siRNAs) keratinocytes. Data are shown as mean ± SD of N=3 biological replicates. The adjusted p-values were calculated using One-way Anova. **B)** The proteins expression of p63 was evaluated using western blot in transfected (Ctrl, sip63#1 and sip63#2 siRNAs) keratinocytes. GAPDH served as loading control. **C)** The Protein expression of p63 and p16 was evaluated using western blot in senescent (P1, P2, P3 and P4) keratinocytes. β-actin served as loading control. **D)** The proteins expression of p63, p53 and p16 was evaluated using western blot in senescent (P1 and P4) keratinocytes. β-actin served as loading control. **E)** The mRNA expression level of *GGT1, GCLC, GSS*, *GPX1* and *OPLAH* were evaluated using qRT-PCR in transfected (Ctrl, sip63#1 and sip63#2 siRNAs) keratinocytes. Data are shown as mean ± SD of N=3 biological replicates. The adjusted p-values were calculated using Student's t-test. n.s.=non significant. **F)** The mRNA expression level of *GGT5*, *GSR, GSS* and *OPLAH* were evaluated using qRT-PCR in senescent (P1, P4) keratinocytes. Data are shown as mean ± SD of N=3 biological replicates. The adjusted p-values were calculated using Student's t-test. n.s.=non significant.

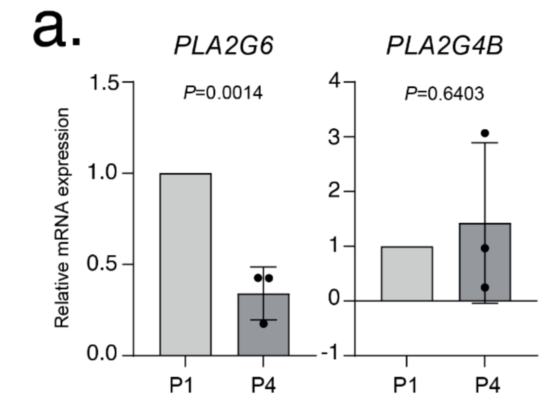

Fig. S2. Altered glycerophospholipid metabolism. A) The mRNA expression level of *PLA2G6, PLA2G4B* were evaluated using qRT-PCR in senescent (P1, P4) keratinocytes. Data are shown as mean ± SD of N=3 biological replicates. The adjusted p-values were calculated using Student's t-test. n.s.=non significant.

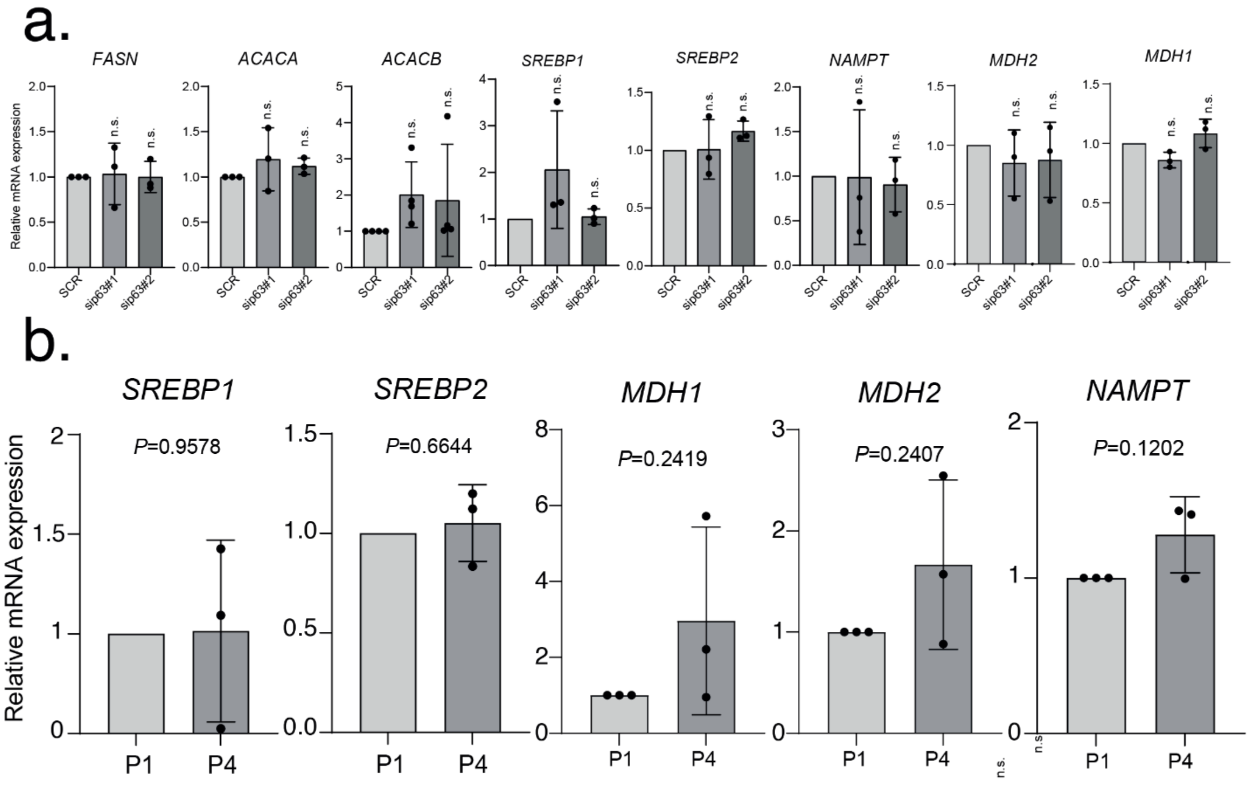

Fig. S3. Increased fatty acids metabolism. A) The mRNA expression level of *FASN, ACACA, ACACB, SREBP1* and *SREBP2, NAMPT, MDH2 and MDH1* were evaluated using qRT-PCR in transfected (Ctrl, sip63#1 and sip63#2 siRNAs) keratinocytes. Data are shown as mean ± SD of N>3 biological replicates. The adjusted p-values were calculated using Student's t-test. n.s.=non significant. **B)** The mRNA expression level of *SREBP1, SREBP2, MDH1, MDH2,* and *NAMPT* were evaluated using qRT-PCR in senescent (P1, P4) keratinocytes. Data are shown as mean ± SD of N=3 biological replicates. The adjusted p-values were calculated using Student's t-test. n.s.= non significant.

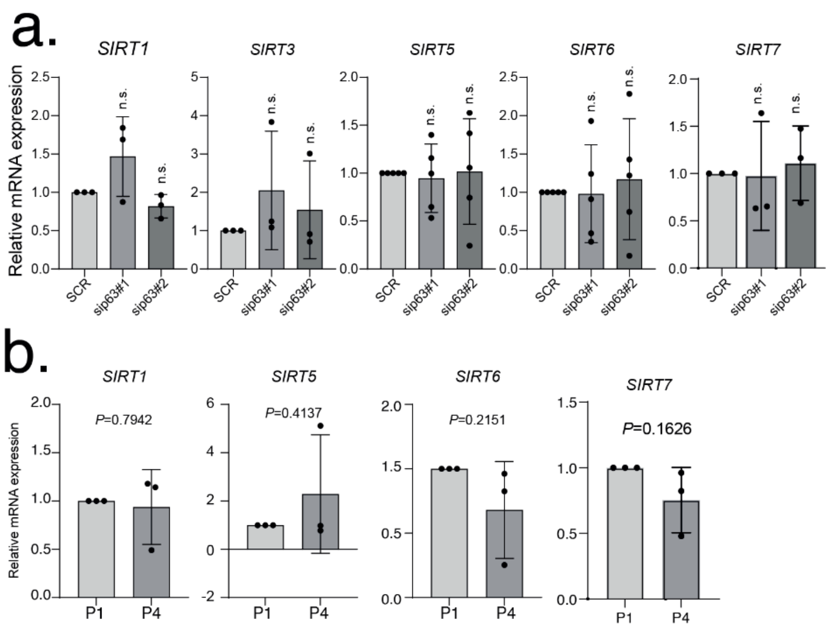

Fig. S4. Sirtuins A) The mRNA expression level of *SIRT1, SIRT3, SIRT5, SIRT6* and *SIRT7* were evaluated using qRT-PCR in transfected (Ctrl, sip63#1 and sip63#2 siRNAs) keratinocytes. Data are shown as mean ± SD of N>3 biological replicates. The adjusted p-values were calculated using Student's t-test. n.s.=non significant. **B)** The mRNA expression level of *SIRT1, SIRT5* and *SIRT6* and *SIRT7* were evaluated using qRT-PCR in senescent (P1, P4) keratinocytes. Data are shown as mean ± SD of N=3 biological replicates. The adjusted p-values were calculated using Student's t-test. n.s.=non significant.

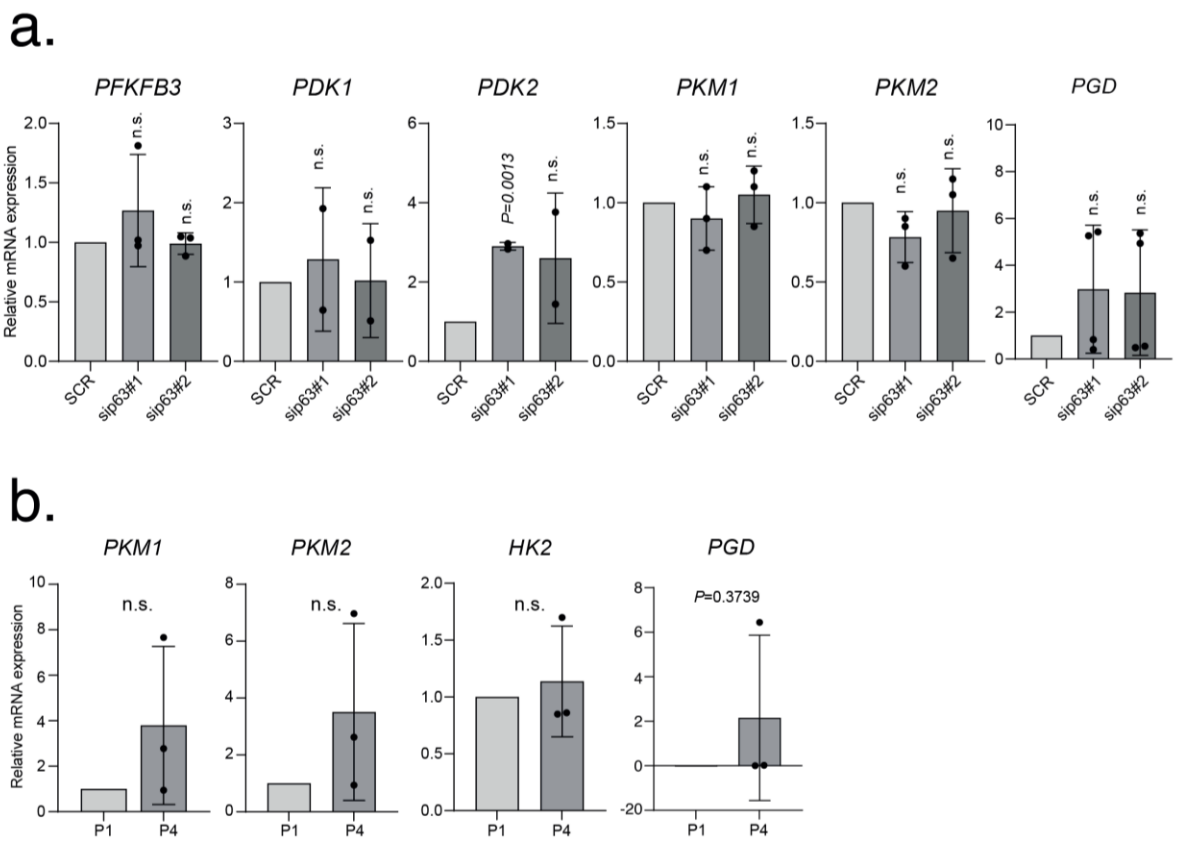

Fig. S5. Altered glucose metabolism. A) The mRNA expression level of *PFKFB3, PDK1, PDK2, PKM1*, *PKM2* and *PGD* were evaluated using qRT-PCR in transfected (Ctrl, sip63#1 and sip63#2 siRNAs) keratinocytes. Data are shown as mean ± SD of N=3 biological replicates. The adjusted p-values were calculated using Student's t-test. n.s= non significant. **B)** The mRNA expression level of *PKM1, PKM2, HK2, PGD,* were evaluated using qRT-PCR in senescent (P1, P4) keratinocytes. Data are shown as mean ± SD of N=3 biological replicates. The adjusted p-values were calculated using Student's t-test. n.s.=non significant.

Materials and methods , Supplementary Material

QA/QC: For QA/QC purposes, a number of additional samples are included with each day's analysis. Furthermore, a selection of QC compounds is added to every sample, including those under test. These compounds are carefully chosen so as not to interfere with the measurement of the endogenous compounds. Tables 1 and 2 describe the QC samples and compounds. These QC samples are primarily used to evaluate the process control for each study as well as aiding in the data curation.

Table 1: Description of Metabolon QC Samples

Type	Description	Purpose
MTRX	Large pool of human plasma maintained by Metabolon that has been characterized extensively.	Assure that all aspects of Metabolon process are operating within specifications.
CMTRX	Pool created by taking a small aliquot from every customer sample.	Assess the effect of a non-plasma matrix on the Metabolon process and distinguish biological variability from process variability.
PRCS	Aliquot of ultra-pure water	Process Blank used to assess the contribution to compound signals from the process.
SOLV	Aliquot of solvents used in extraction.	Solvent blank used to segregate contamination sources in the extraction.

Table 2: Metabolon QC Standards

Type	Description	Purpose
DS	Derivatization Standard	Assess variability of derivatization for GC/MS samples.
IS	Internal Standard	Assess variability and performance of instrument.
RS	Recovery Standard	Assess variability and verify performance of extraction and instrumentation.

Data Quality: Instrument and Process Variability

QC Sample	*Measurement*	*Median RSD*
Internal Standards	Instrument Variability	6 %
Endogenous Biochemicals	Total Process Variability	13 %

Instrument variability was determined by calculating the median relative standard deviation (RSD) for the internal standards that were added to each sample prior to injection into the mass spectrometers. Overall process variability was determined by calculating the median RSD for all endogenous metabolites (i.e., non-instrument standards) present in 100% of the Client Matrix samples, which are technical replicates of pooled client samples. Values for instrument and process variability meet Metabolon's acceptance criteria as shown in the table above.

Bioinformatics: The informatics system consisted of four major components, the Laboratory Information Management System (LIMS), the data extraction and peak-identification software, data processing tools for QC and compound identification, and a collection of information interpretation and visualization tools for use by data analysts. The hardware and software foundations for these informatics components were the LAN backbone, and a database server running Oracle 10.2.0.1 Enterprise Edition.

LIMS: The purpose of the Metabolon LIMS system was to enable fully auditable laboratory automation through a secure, easy to use, and highly specialized system. The scope of the Metabolon LIMS system encompasses sample accessioning, sample preparation and instrumental analysis and reporting and advanced data analysis. All of the subsequent software systems are grounded in the LIMS data structures. It has been modified to leverage and interface with the in-house information extraction and data visualization systems, as well as third party instrumentation and data analysis software.

Data Extraction and Quality Assurance: The data extraction of the raw mass spec data files yielded information that could loaded into a relational database and manipulated without resorting to BLOB manipulation. Once in the database the information was examined and appropriate QC limits were imposed. Peaks were identified using Metabolon's proprietary peak integration software, and component parts were stored in a separate and specifically designed complex data structure.

Compound identification: Compounds were identified by comparison to library entries of purified standards or recurrent unknown entities. Identification of known chemical entities was based on comparison to metabolomic library entries of purified standards. As of this writing, more than 1000 commercially available purified standard compounds had been acquired registered into LIMS for distribution to both the LC and GC platforms for determination of their analytical characteristics. The combination of chromatographic properties and mass spectra gave an indication of a match to the specific compound or an isobaric entity. Additional entities could be identified by virtue of their recurrent nature (both chromatographic and mass spectral). These compounds have the potential to be identified by future acquisition of a matching purified standard or by classical structural analysis.

Curation: A variety of curation procedures were carried out to ensure that a high quality data set was made available for statistical analysis and data interpretation. The QC and curation processes were designed to ensure accurate and consistent identification of true chemical entities, and to remove those representing system artifacts, mis-assignments, and background noise.

Metabolon data analysts use proprietary visualization and interpretation software to confirm the consistency of peak identification among the various samples. Library matches for each compound were checked for each sample and corrected if necessary.

Normalization: For studies spanning multiple days, a data normalization step was performed to correct variation resulting from instrument inter-day tuning differences. Essentially, each compound was corrected in run-day blocks by registering the medians to equal one (1.00) and normalizing each data point proportionately (termed the "block correction"; Figure 1). For studies that did not require more than one day of analysis, no normalization is necessary, other than for purposes of data visualization.

Figure 1: Visualization of Data Normalization

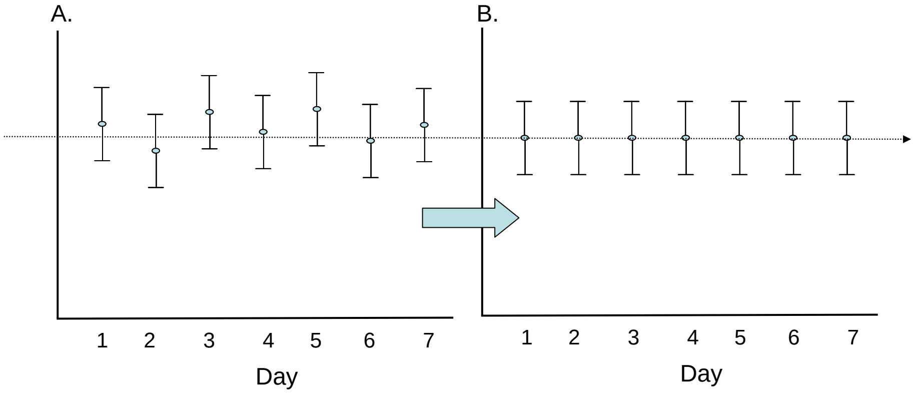

Statistical Calculation: For many studies, two types of statistical analysis are usually performed: (1) significance tests and (2) classification analysis. (1) For pair-wise comparisons we typically perform Welch's t-tests and/or Wilcoxon's rank sum tests. For other statistical designs we may perform various ANOVA procedures (e.g., repeated measures ANOVA). (2) For classification we mainly use random forest analyses. Random forests give an estimate of how well we can classify *individuals* in a *new* data set into each group, in contrast to a t-test, which tests whether the unknown means for two populations are different or not. Random forests create a set of classification trees based on continual sampling of the experimental units and compounds. Then each observation is classified based on the majority votes from all the classification trees. Statistical analyses are performed with the program "R" http://cran.r-project.org/.

Figure S1 b.

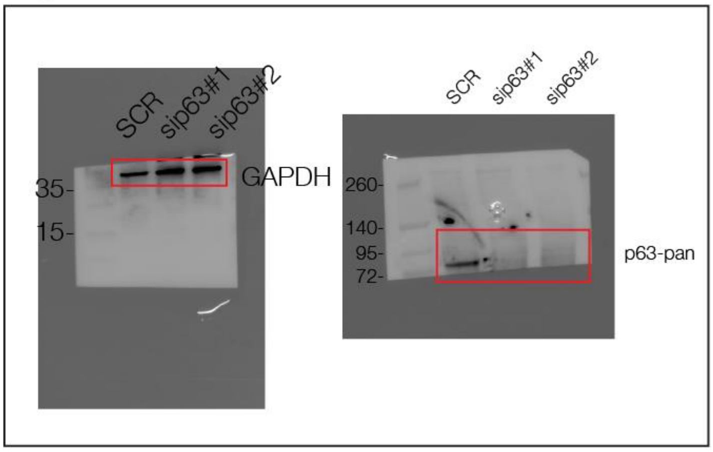

Figure S1 c.

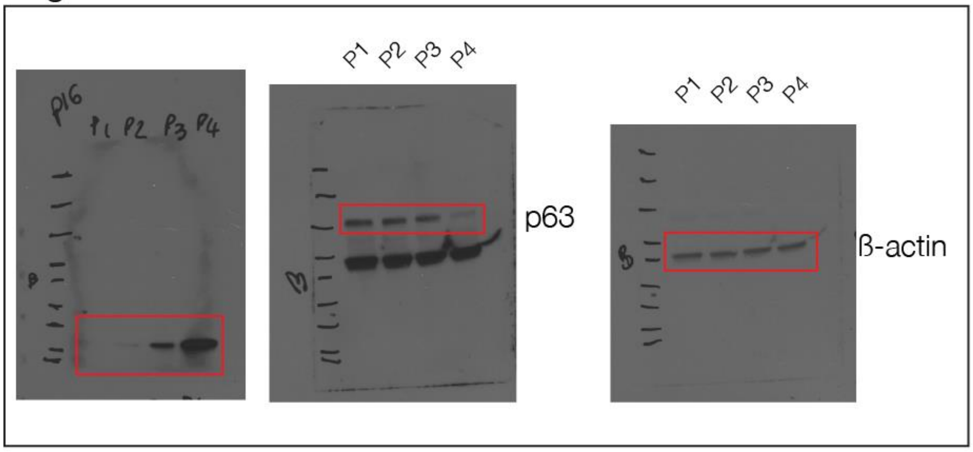

Figure S1 d.

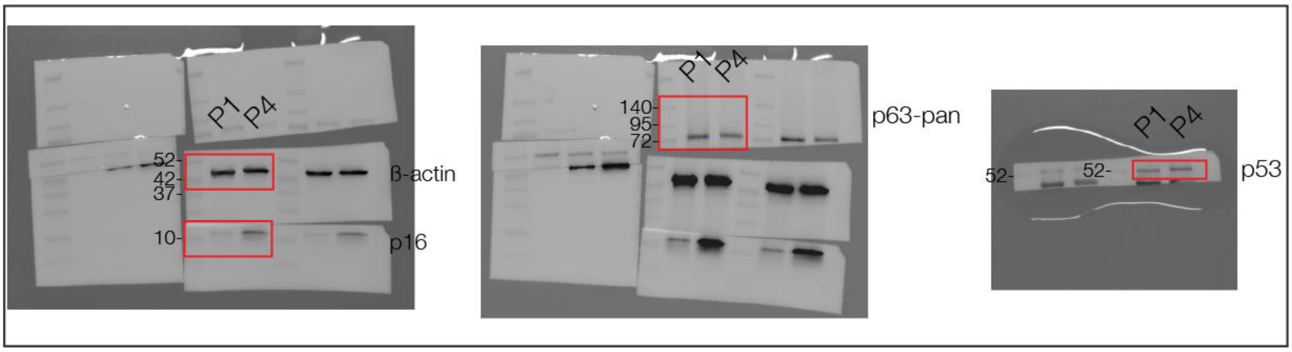

Supplemental material. Original blot

**Table S1** Primers list		
**GENE**	**Forward Primer**	**Reverse Primer**
ACACA	TTCACTCCACCTTGTCAGCGGA	GTCAGAGAAGCAGCCCATCACT
ACACB	GACGAGCTGATCTCCATCCTCA	ATGGACTCCACCTGGTTATGCC
ACLY	GCTCTGCCTATGACAGCACCAT	GTCCGATGATGGTCACTCCCTT
CS	CACAGGGTATCAGCCGAACCAA	CCAATACCGCTGCCTTCTCTGT
FASN	TTCTACGGCTCCACGCTCTTCC	GAAGAGTCTTCGTCAGCCAGGA
G6PD	CTGTTCCGTGAGGACCAGATCT	TGAAGGTGAGGATAACGCAGGC
GCLC	GGAAGTGGATGTGGACACCAGA	GCTTGTAGTCAGGATGGTTTGCG
GGT1	TGACGTACCACCGCATCGTAGA	CAGCGAAGAACTCGGAGGTCAT
GGT5	CAGATGCTGGTGGAGGACATTG	GTGGTGAGTACAGGGTATAGTCC
GGT6	GACATCAGAGGCGCTGGTTCTA	CAGCAGGGCTGCCACTACACG
GGT7	GGACTTCAGCAATTACAGCGCC	CTCCAGGATGTTGAGAGCACTG
GPX1	ACGATGTTGCCTGGAACTTT	TCGATGTCAATGGTCTGGAA
GPX2	GTGCTGATTGAGAATGTGGC	AGGATGCTCGTTCTGCCCA
GSR	TATGTGAGCCGCCTGAATGCCA	CACTGACCTCTATTGTGGGCTTG
GSS	CCAAGACCGAAGGCTGTTTGTG	TGTGACCTCTCCAGCAGTAGAC
GSTP1	TGGACATGGTGAATGACGGCGT	GGTCTCAAAAGGCTTCAGTTGCC
HK2	GGTTTTACCTTCTCGTTCCCCTGC	TCAAAGTCCCCTCTCCTCTGGATG
MDH1	CGGTGTCCTAATGGAACTGCAAG	CATCCAGGTCTTTGAAGGCAACG
MDH2	CTGGACATCGTCAGAGCCAACA	GGATGATGGTCTTCCCAGCATG
NAMPT	AGGGTTACAAGTTGCTGCCACC	CTCCACCAGAACCGAAGGCAAT
OPLAH	GACTGTGCTCTGATGGTGTCTG	CCGCTCAGGTATGACAAAGCCA
PDK1	CATGTCACGCTGGGTAATGAGG	CTCAACACGAGGTCTTGGTGCA
PDK2	TGCCTACGACATGGCTAAGCTC	GACGTAGACCATGTGAATCGGC
PDK3	TGGAAGGAGTGGGTACTGATGC	GGATTGCTCCAATCATCGGCTTC
PDK4	AGGTGGAGCATTTCTCGCGCTA	GAATGTTGGCGAGTCTCACAGG
PFKFB3	GGCAGGAGAATGTGCTGGTCAT	CATAAGCGACAGGCGTCAGTTTC
PGD	GTTCCAAGACACCGATGGCAAAC	CACCGAGCAAAGACAGCTTCTC
PKM1	CGAGCCTCAAGTCACTCCAC	GTGAGCAGACCTGCCAGACT
PKM2	ATGGCTGACACATTCCTGGAGC	CCTTCAACGTCTCCACTGATCG
PLA2G16	GAGGTGCTCTACAAGCTGACCA	CTCCAATAAGGCTCATGGCTGC
PLA2G1B	ACAACTACGGCTGCTACTGTGG	GTGTACGGGTTGTCCAGCAGAA
PLA2G4A	GGATTCTCTGGTGTGATGAAGGC	CCTTTCTCTGGAAAATCAGGGTG
PLA2G4B	CCGCTCAGGTATGACAAAGCCA	CCAGCTTGTTCTTGGTCACCTG
PLA2G6	CCTCATCGTGTTCGGAGCAGAA	CACGGTTCTCAGCAGAGTCAAG
PNPLA1	CAGTCTGGAAGGAGCCACACAA	GCTGAAACAGGTGACTCGCATG
SIRT1	TAGACACGCTGGAACAGGTTGC	CTCCTCGTACAGCTTCACAGTC
SIRT2	CTGCGGAACTTATTCTCCCAGAC	CCACCAAACAGATGACTCTGCG
SIRT3	CCCTGGAAACTACAAGCCCAAC	GCAGAGGCAAAGGTTCCATGAG
SIRT4	GTGGATGCTTTGCACACCAAGG	GGTTCAGGACTTGGAAACGCTC
SIRT5	GTCCACACGAAACCAGATTTGCC	TCCTCTGAAGGTCGGAACACCA
SIRT6	TGGCAGTCTTCCAGTGTGGTGT	CGCTCTCAAAGGTGGTGTCGAA
SIRT7	TGGAGTGTGGACACTGCTTCAG	CCGTCACAGTTCTGAGACACCA
SREBP1 (SREBF1)	ACTTCTGGAGGCATCGCAAGCA	AGGTTCCAGAGGAGGCTACAAG
SREBP2 (SREBF2)	CTCCATTGACTCTGAGCCAGGA	GAATCCGTGAGCGGTCTACCAT
TAp63	TCAGAAGATGGTGCGACAAAC	GTTCAGGAGCCCCAGGTTCG
ΔNp63	GAAGAAAGGACAGCAGCATTG	GGGACTGGTGGACGAGGAG
p73	CAGACAGCACCTACTTCGACCTT	CCGCCCACCACCTCATTA
TBP	TCAAACCCAGAATTGTTCTCCTTAT	CCTGAATCCCTTTAGAATAGGGTAGA
